# LysR-type transcriptional regulator TtdR regulates both ethylene glycol and polyhydroxyalkanoate (PHA) metabolism in *Pseudomonas umsongensis* GO16

**DOI:** 10.1007/s00253-026-13865-3

**Published:** 2026-05-19

**Authors:** Jounghyun Um, Karthika Balusamy, Nick Wierckx, Kevin E. O’Connor, Tanja Narancic

**Affiliations:** 1 UCD Earth Institute and School of Biomolecular and Biomedical Science, University College Dublin, Belfield, Dublin 4, Ireland; 2 BiOrbic - Bioeconomy Research Centre, University College Dublin, Belfield, Dublin 4, Ireland; 3 Institute of Bio- and Geosciences IBG-1: Biotechnology, Forschungszentrum Jülich, Jülich, Germany

**Keywords:** *Pseudomonas umsongensis* GO16, Ethylene glycol, Glyoxylate, LysR-type transcriptional regulator, Polyhydroxyalkanoates

## Abstract

In *Pseudomonas umsongensis* GO16, a LysR-type transcriptional regulator (LTTRs) *ttdR* is located 5′ of the gene for glyoxylate carboligase (*gcl*), involved in ethylene glycol (EG) metabolism. GO16 wildtype (WT) can utilize EG as a sole carbon and energy source but the *ttdR* knockout strain (Δ*ttdR*) cannot grow on EG, demonstrating its role as an activator in EG metabolism. When Δ*ttdR* was grown with terephthalic acid (TA) or glucose, the growth was comparable to the WT. GO16 originally produces both C4 short-chain-length (scl) PHA and C6-C12 medium-chain-length (mcl) PHA from various carbon sources. Interestingly, Δ*ttdR* produced scl-PHA monomer 5.4-fold and 1.4-fold increased than the WT when TA and glucose were used respectively. Furthermore, Δ*ttdR* exhibited a very long lag phase when grown with fatty acids, namely butyrate or octanoate. This indicates a more complex role of TtdR than simply activating EG metabolism in GO16. The analysis of the GO16 Δ*ttdR* proteome revealed that EG catabolic genes were expressed, but their abundance was 2.8- to 10.5-fold lower (log_2_) compared to the WT. In addition, higher abundance of enzymes that could be contributing to higher scl-PHA content was also observed. GO16 Δ*ttdR* was further subjected to adaptive laboratory evolution (ALE) with butyrate and octanoate. The evolved strains regained the ability to grow with these fatty acids as the WT, but the mutations accumulated did not confer growth with EG or acetate, suggesting that a mechanism different to glyoxylate shunt has evolved to allow growth with fatty acids.

**Supplementary Information:**

The online version contains supplementary material available at 10.1007/s00253-026-13865-3.

## Highlights

- *A LysR-type transcriptional regulator, TtdR, activates ethylene glycol (EG) metabolism.*
- *Deletion of TtdR impairs fatty acid utilization and increases short-chain-length polyhydroxyalkanoate (PHA) accumulation.*
- *Proteomic analysis and adaptive laboratory evolution (ALE) were performed to investigate the role of TtdR as a global regulator influencing multiple pathways, including EG metabolism, fatty acid utilization, and PHA synthesis.*

**Graphical abstract:**

A LysR-type transcriptional regulator, TtdR, is located upstream (5′) of the glyoxylate carboligase gene (*gcl*) on the chromosome of *P. umsongensis* GO16. Deletion of *ttdR* resulted in no growth on ethylene glycol and delayed growth on fatty acids such as butyrate and octanoate. The strain showed comparable growth on terephthalic acid and glucose to the wildtype GO16 but exhibited increased synthesis of short-chain-length PHA (C4) monomer. This suggests that TtdR is an activator for ethylene glycol metabolism and also influences fatty acid and PHA metabolism.

**Supplementary Information:**

The online version contains supplementary material available at 10.1007/s00253-026-13865-3.

## Graphical abstract

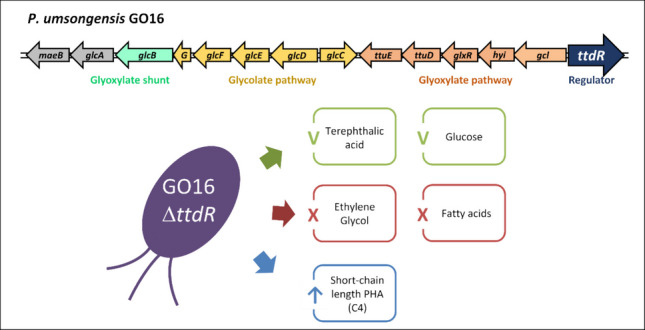

## Introduction

Ethylene glycol (EG) is an important molecule which is commonly used in various industrial applications, including as a raw material to synthesize polyethylene terephthalate (PET) and in antifreeze solutions (Cen et al. 2022). Its global consumption is 31.93 million metric tons (Mt) in 2022 and is expected to increase to 42 Mt in the coming decades (Statista 2023).

**Fig. 1:**
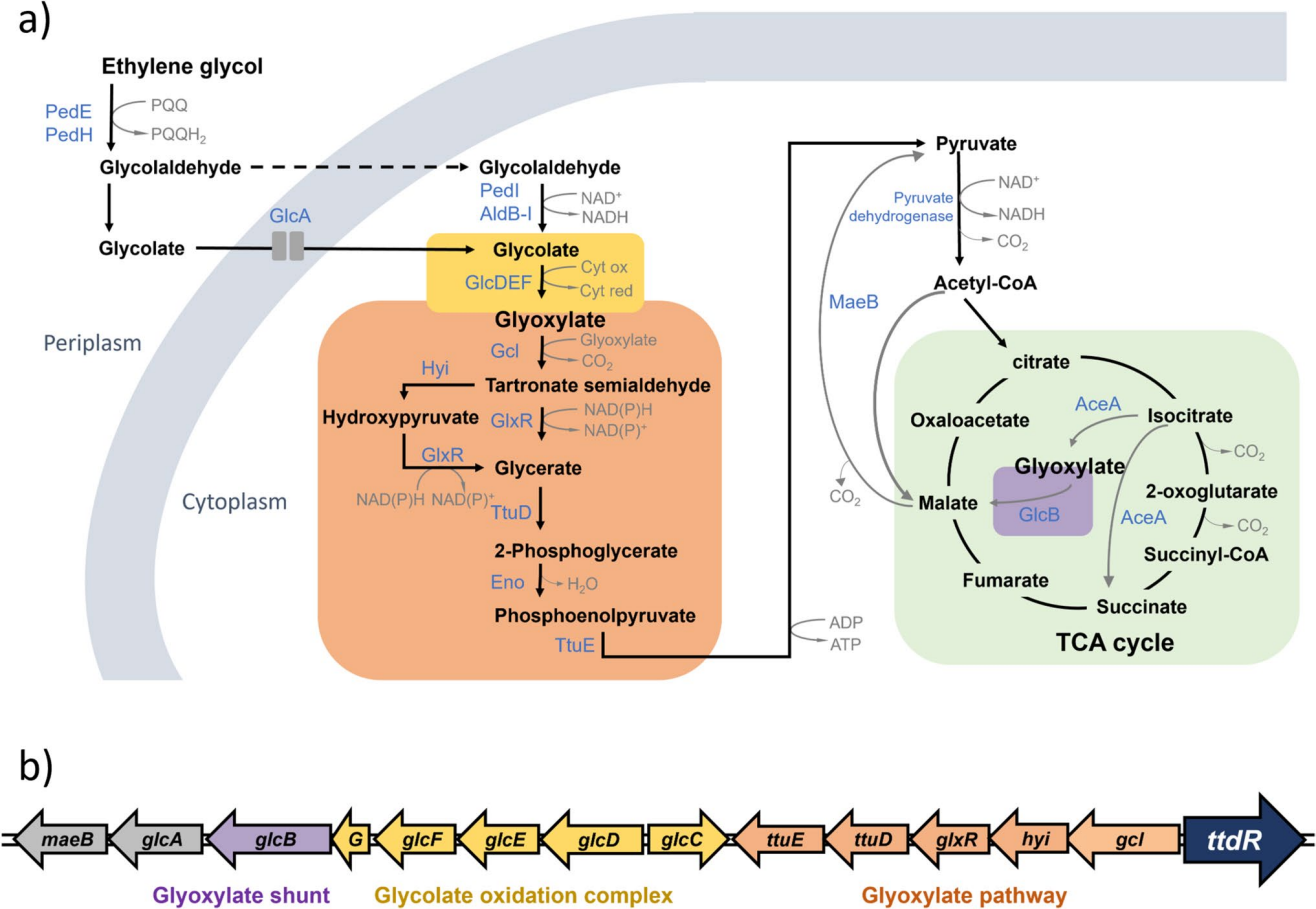
Overview of EG metabolism and gene organization in *P. umsongensis* GO16. **a** Schematic representation of EG metabolism and its integration into central carbon metabolism in *Pseudomonas* species*.* The colors of the labels indicate enzymes (blue), metabolites (black), and cofactors and small molecules (gray). The color-coded metabolic routes, including the glycolate oxidation complex (yellow), glyoxylate pathway (orange), glyoxylate shunt (purple), and TCA cycle (green), correspond directly to the gene clusters shown in **b**. The genes encoding PedH and PedE, essential for the initial steps of EG oxidation, are not depicted as they are not part of the EG cluster: *pedH* is located 3,947,017 bp from the 3*′* end of *ttdR*, and *pedE* is located 3577 bp further downstream from the 3*′* end of *pedH*. PedEH, quinoprotein alcohol dehydrogenases; PedI and AldB-I, aldehyde dehydrogenases; GlcDEF, glycolate oxidase; AceA, isocitrate lyase; GlcB, malate synthase; Gcl, glyoxylate carboligase; Hyi, hydroxypyruvate isomerase; GlxR, tartronate semialdehyde reductase; TtuD, hydroxypyruvate reductase; Eno, phosphopyruvate hydratase; TtuE, pyruvate kinase; PDH, pyruvate dehydrogenase; MaeB, malate dehydrogenase; OAA, oxaloacetate; PEP, phosphoenolpyruvate

It was shown that EG can serve as a carbon and energy source for some bacteria, such as *Flavobacterium* sp., *Mycobacterium* E44, *Ideonella sakaiensis*, *Pseudomonas putida* JM37 (Child and Willetts 1978; Gaston and Stadtman 1963; Hachisuka et al. 2022; Trifunović et al. 2016; Wiegant and De Bont 1980; Willetts 1981). In *P. putida* JM37, the catabolism of EG begins with PedH and PedE, pyrroloquinoline quinone (PQQ)-dependent broad substrate range alcohol dehydrogenases (Fig. 1a) (Wehrmann et al. 2017). The resulting glycolaldehyde is further oxidized by cytoplasmic aldehyde dehydrogenases AldB-I and PedI, and a membrane-anchored oxidase GlcDEF, finally producing glyoxylate through glycolaldehyde and glycolate intermediates. The critical reactions enabling the conversion of EG to biomass are the conversion of two glyoxylate molecules to tartronate semialdehyde and CO_2_ through a glyoxylate carboligase (Gcl) and subsequent conversion to 2-phosphoglycerate which enters the central metabolic pathways.

While the complement of EG catabolic genes was found in *P. putida* KT2440, this strain was only able to use this substrate for growth when Gcl in combination with other genes related to glyoxylate metabolism was overexpressed using a strong, constitutive promoter (Franden et al. 2018). In a companion study, Li and co-workers obtained a KT2440 strain through adaptive laboratory evolution (ALE) that could grow with EG as a sole carbon and energy source, due to a mutation in the *gclR* gene, rendering this repressor inactive (Li et al. 2019).

Homologous EG catabolic genes were found in the genome of *P. umsongensis* GO16, suggesting its EG metabolic pathway is similar to that of other *Pseudomonas* species (Fig. 1a). However, the genomic organization of EG catabolic genes in GO16 is distinct, featuring a combined cluster of glyoxylate carboligase and glyoxylate cycle genes located nearby. This arrangement, which differs significantly from that observed in *Pseudomonas putida*, suggests that the metabolic flux from glyoxylate to malate, pyruvate, and subsequently to acetyl-CoA may be the predominant route in GO16. In addition, the *gcl* gene cluster (the “glyoxylate pathway,” Fig. 1b) is preceded by a LysR-type transcriptional regulator (LTTR) TtdR (F6476_RS01225), not a GntR-type regulator GclR observed in *P. putida* KT2440 (Narancic et al. 2021).

TtdR has a signature helix-turn-helix (HTH) motif at the N-terminus from amino acids 6 to 63 and a substrate binding domain at the C-terminal from amino acids 92 to 289. Its total size is 306 amino acids, which is consistent with proteins in this family of more than 40,000 regulators involved in signal-dependent and signal-independent transcriptional regulation (Reen et al. 2013). They can either activate or repress transcription of target genes, including those involved in virulence, stress response, catabolism of aromatic compounds, and others. Some of the LTTRs have a role as a global regulator, which gives an advantage of minimizing the need for multiple regulatory proteins (Maddocks and Oyston 2008; Reen et al. 2013).

The role of GO16 in upcycling petrochemical plastic PET was demonstrated (Kenny et al. 2008; Narancic et al. 2021; Orimaco et al. 2025). Furthermore, GO16 can synthesize both short-chain-length (scl) and medium-chain-length (mcl) polyhydroxyalkanoates (PHA), a class of biodegradable polyesters (Cerrone et al. 2023; Narancic et al. 2021). Typical pathways, i.e., scl-PHA synthesis pathway, or de novo fatty acid synthesis and *β*-oxidation, are employed for PHA synthesis (Mozejko-Ciesielska et al. 2019; Peplinski et al. 2010; Sagong et al. 2018). The advantage of using GO16 and its native capacity to blend scl- and mcl- lies in the fact that the blend has improved viscoelasticity compared to the neat polymers (Cerrone et al. 2023).

To further enhance the potential of GO16 as a catalyst for the conversion of post-consumer PET into PHA and other valuable molecules, we investigated the role of TtdR in GO16. The deletion strain GO16 Δ*ttdR* lost the capacity to use EG as a carbon and energy source, suggesting that TtdR is actively regulating a part of EG metabolism in GO16. Off-target effects were also observed. GO16 Δ*ttdR* had a prolonged lag phase when fatty acids, butyrate, or octanoate were used as substrates, and the deletion strain showed an increased scl-PHA fraction when grown with substrates such as glucose or terephthalic acid (TA). This indicates that TtdR may have a more global role. To understand which specific targets may be regulated by TtdR, we carried out adaptive laboratory evolution (ALE) using butyrate or octanoate as substrates.

## Materials and methods

### Strains and culture conditions

All strains (Table 1) were maintained in Lysogeny Broth (LB) or Minimal Salts Medium (MSM; (Orimaco et al. 2025)) with 30% glycerol at −70 °C freezer. *E. coli* strains were cultivated on LB medium with the relevant antibiotic at 37 °C and 200-rpm incubator, and *Pseudomonas* strains were cultured on MSM medium. MSM_full_ and MSM_lim_ were used to evaluate how nutrient availability influences bacterial growth and PHA accumulation. One gram per liter of NH_4_Cl was added for the MSM_full_ condition, which contains sufficient nitrogen to support active cell growth and biomass production. In contrast, 0.25 g L^−1^ of NH_4_Cl was added for MSM_lim_ which provides nitrogen-limiting conditions while maintaining carbon availability, a metabolic state known to suppress further biomass formation and redirect excess carbon toward intracellular storage polymer synthesis such as PHA. Carbon sources were added to a final carbon concentration of 1.96 g_c_ L^−1^, e.g., 20 mM TA, 26.5 mM glucose, 20 mM octanoate, 40 mM butyrate, or 82 mM EG, unless otherwise stated. When TA and EG were co-fed, 20 mM TA and 30 mM EG were added to the medium.

**Table 1:** List of bacterial strains used in this study

Strain	Description	Source
GO16 WT	*Pseudomonas umsongensis* GO16 wildtype strain; accession number NCIMB 41538, NCIMB Aberdeen, Scotland, UK	Kenny et al. (2008)
GO16 Δ*ttdR*	*ttdR* (F6476_RS01225) knocked out from GO16 WT	This study
GO16 pBT′T_*ttdR*	GO16 WT bearing *ttdR* expressed in pBT′T plasmid, Kan^R^	This study
GO16 pBT′T _eYFP	GO16 WT containing pBT′T_eYFP (green/yellow fluorescent protein), Kan^R^	This study
GO16 Δ*ttdR_*ALE_But_	Adaptive laboratory evolution (ALE) strain of Δ*ttdR* cultivated on MSM Butyrate by serial dilution until a similar growth to GO16 WT was achieved	This study
GO16 Δ*ttdR_*ALE_Oct_	Adaptive laboratory evolution (ALE) strain of Δ*ttdR* cultivated on MSM Octanoate by serial dilution until a similar growth to GO16 WT was achieved	This study
*E. coli* DH5α	General cloning strain; *endA1 recA1 φ80dlacZΔM15*	Novagen
*E. coli* DH5α-λpir	General cloning strain; *endA1 hsdR17 glnV44 (*= *supE44) thi-1 recA1 gyrA96 relA1 φ80dlacΔ(lacZ)M15* Δ*(lacZYA-argF)U169 zdg-232::Tn10 uidA::pir* +	Novagen
*P. putida* KT2440 pCas9/λRed	*P. putida* KT2440 containing a pCas9/λRed Recombineering plasmid with constitutively expressed Cas9 and the *araBAD* promoter expressing αβγ, Gm^R^	Cook et al. (2018)
*E. coli* DH5α-λpir pKnock	*E. coli* DH5α-λpir bearing a suicide plasmid for gene knockout, Kan^R^	Addgene
*E. coli* DH5α pgRNA	*E. coli* DH5α-λpir containing a guide RNA plasmid, Tet^R^	Cook et al. (2018)

The inoculum was brought to inoculate fresh medium with an OD_540nm_ of 0.05. Small-scale growth experiments using BioLector (Flowerplate MTP 48-well, BioLector® I, m2p-labs GmbH, Germany) were carried out in 1 mL of MSM_full_ medium with corresponding carbon source and trace elements at 30 °C and 1,000 rpm. The experiment was performed using three independent biological replicates, with each sample analyzed in technical triplicates.

Strains were also tested in 250-mL Erlenmeyer flasks containing 50 mL MSM_full_ or MSM_lim_ supplemented with carbon sources and trace elements at 30 °C and 200 rpm for 48 h or more depending on the measured OD_540nm_. Three independent biological replicates, defined as distinct colonies selected from a single agar plate, were utilized. Statistical significance and data dispersion are represented in all figures as the Standard Deviation (SD).

### Generation of *P. umsongensis* GO16 Δ*ttdR*

The knockout of *ttdR* was scarlessly generated using a CRISPR/Cas9 system and methodology (Cook et al. 2018; Liu et al. 2022). The primers and DNA sequence of the single guide RNA (sgRNA) employed for knockout are detailed in supplementary data (Table. S1). The sgRNA was designed using EuPaGDT CRISPR guide RNA/DNA design tool (http://grna.ctegd.uga.edu/) to target the sequence of the *ttdR* gene to be deleted.

### TtdR over-expressing plasmid construction

The *ttdR* was amplified by PCR and cloned into the expression plasmid pBT′T (Koopman et al. 2010) using NEBuilder® HiFi DNA Assembly Master Mix (NEB, UK) as recommended by the manufacturer. Two microliters of the Gibson reaction was transferred into *E. coli* DH5α by heat shock transformation. The pBT′T_*ttdR* construct was verified by sequencing (Eurofins, Ireland) and transformed into *P. umsongensis* GO16 by electroporation (Choi et al. 2006). The transformants were selected on MSM_full_ agar plate with 20 mM TA and 50 µg mL^−1^ of kanamycin and confirmed by colony PCR using primers. The details of primers are listed in the supplementary data (Table S2).

### Biomass (cell dry weight; CDW) analysis

Forty-eight-hour cultures were harvested by centrifugation at 6000 g for 10 min at 4 °C. Two milliliters of supernatant was kept at −20 °C to analyze nitrogen and carbon consumption. Cell pellets were washed twice with 10 mL of 100 mM phosphate-buffered saline (PBS, pH 7.4), resuspended in 1 mL 100 mM PBS and transferred into 2-mL microcentrifuge tubes. The 2-mL tubes were centrifuged at 14,500 rpm for 5 min at room temperature. Supernatant was removed and the pellets were kept at −70 °C for freeze-drying. The frozen cell pellets were lyophilized using Labconco FreeZone 12 (USA) for 24 h and weighed.

### PHA quantification

The PHA content was extracted using acidic methanolysis of 5–15 mg of lyophilized cells as previously described (Lageveen et al. 1988). The resultant 3-hydroxyalkanoic acid (R3HA) methyl esters were analyzed by a 6890 N Gas Chromatogram (Agilent Technologies, UK) equipped with a HP-Innowax Capillary column (30 m × 0.25 mm × 0.5 µm, Agilent Technologies, USA) using a split mode (split ratio 10:1) and a flame ionization detector (FID). The oven was heated to 120 °C for 5 min, then increased at a rate of 5 °C/min until it reached 180 °C, and maintained for 10 min. The peak was identified by commercially available R3HA methyl esters (Bioplastech, Ireland). To validate the efficiency of the procedure, a recovery study was performed using a pure PHA standard (Merck, Ireland), yielding an average recovery rate of 85%. All experimental data were subsequently adjusted using this recovery coefficient to account for analytical losses during the transesterification and phase separation stages.

### Carbon consumption

The consumption of TA was analyzed by a 1260 Infinity II HPLC (Agilent, USA) using a C18 ODS-Hypersil Column (125 mm × 3 mm × 5 µm, Thermo Scientific, USA) with 10-times diluted supernatant collected after the cultivation. Samples were eluted using 0.4% formic acid and 100% acetonitrile at a flow rate of 1 mL min^−1^ and read using a diode-array detector (DAD). EG and glucose depletion were monitored on a Shimadzu HPLC using an Aminex HPX-87H ion exclusion column (300 mm × 7.8 mm × 9 µm, Bio-Rad, UK). The column was maintained at 40 °C and samples were isocratically eluted using 0.014 N H_2_SO_4_ at a flow rate of 0.55 mL min^−1^ and read on a refractive index detector (RID).

### Proteomics analysis

Cell cultures under PHA accumulating or non-accumulating conditions supplemented with TA, glucose, or TA and EG were collected at 24 and 48 h. Cell pellets were resuspended in 8 M urea, treated with trypsin and purified as previously described (Kelly et al. 2024; Wiśniewski et al. 2009). Peptide samples were introduced to the timsTOF Pro mass spectrometer as described (Vasilopoulou et al. 2020). Data were searched with MaxQuant (version 2.0.3.0) (Cox et al. 2014; Cox and Mann 2008) against the redundant Uniprot proteome *Pseudomonas umsongensis* GO16 (UP000326534; 6427 proteins; downloaded 16–11–2022). Trypsin was selected as the enzyme allowing up to two missed cleavages. Variable modifications of oxidation on methionine and N-terminal acetylation as well as the fixed modification of carbamidomethylation of cysteine were included. The Match between Runs (MBR) and Label Free Quantitation (LFQ) options were also selected. The result files from Maxquant were analyzed and visualized by amica (https://github.com/tbaccata/amica) (Didusch et al. 2022).

Proteins were considered presented if they had a minimum of 2 Razor + unique peptides, at least 3 MS/MS counts, and valid values in all triplicates. LFQ intensities were log_2_-transformed, and missing values imputed from a normal distribution shifted 1.8 standard deviations below the mean with a width of 0.3 standard deviations. Differential expression was analyzed using limma (Ritchie et al. 2015). Significant proteins that were more- or less-abundant were extracted from group comparison (GO16.NF.24 h vs Δ*ttdR*.NF.24 h, GO16.NF.48 h vs Δ*ttdR*.NF.48 h, GO16.NL.24 h vs Δ*ttdR*.NL.24 h, GO16.NL.48 h vs Δ*ttdR*.NL.48 h) using log_2_ fold change thresholds greater than 1.5 and an adjusted p-value threshold of 0.05 (S5A), in accordance with the amica guideline. An adjusted p-value is a modified p-value to control the false positive rate (Aguilan et al. 2020; Louie et al. 2010). The proteomics datasets generated and analyzed in this study were deposited in the PRIDE partner repository of the ProteomeXchange Consortium and are publicly available under the accession numbers PXD075284 and PXD075327.

### Adapted laboratory evolution (ALE)

ALE was carried out on *P. umsongensis* GO16 Δ*ttdR* strain to improve the growth with butyrate or octanoate. After 120 h of cultivation with butyrate or octanoate, when OD_540nm_ reached over 10 and 13 respectively, the cultures were sampled and streaked onto MSM_full_ agar plates supplemented with 20 mM TA to isolate single colonies. Three single colonies for each substrate were inoculated into three 50 mL tubes (Sarstedt, Germany), each containing 10 mL of MSM_full_ with 20 mM TA, and incubated at 30 °C, 200 rpm. The overnight cultures were transferred into 250-mL Erlenmeyer flasks containing 50 mL MSM_full_, with either 40 mM butyrate or 20 mM octanoate (initial OD_540nm_ = 0.1). The flasks were cultivated until an increase in turbidity (OD_540nm_) was observed. The bacteria were then re-inoculated into fresh media, starting with an OD_540nm_ of 0.05, and incubated as above, and the process was repeated two more times. The 4th passage culture was used to obtain individual cultures by streaking on MSM_full_ agar plates supplemented with 40 mM butyrate or 20 mM octanoate. The three colonies were then assessed for growth and PHA accumulation and sequenced by Azenta/Genewiz (Leipzig, Germany). DNA library preparation, library QC, and sequencing was done on Illumina 2 × 150 bp configuration. The data was analyzed against the reference genome (*P. umsongensis* GO16 chromosome DNA Genbank accession: CP044409.1, Plasmid pENK22: CP044408.1) using sequence analysis tool from Geneious (https://www.geneious.com/).

## Results

### Deletion of LysR-type regulator TtdR abolishes growth on EG

In KT2440, a GclR regulator (PP_4283) from GntR family is a repressor positioned 12 kb upstream from the PP_4297 to the PP_4301 gene cluster involved in EG metabolism (Li et al. 2019). In addition, PP_4283 is connected to xanthine metabolism. This repressor shows 89% amino acid identity with a GntR regulator in GO16 F6476_RS21810, which is 4445 kb apart from the EG gene cluster but closely positioned to the genes involved in xanthine metabolism. The deletion of F6476_RS21810 had no effect on the growth of GO16 with EG, TA, or glucose (Fig. S1).

GO16 has two genes annotated as *gcl*: F6476_RS01220 and F6476_RS05835, showing 85% amino acid identity. F6476_RS01220 precedes *hyi*, *glxR*, *ttuD*, and *ttuE* genes which encode enzymes that catalyze the conversion of glyoxylate to pyruvate. It is followed by the *glcCDEF* operon, which encodes enzymes that convert glycolate into glyoxylate (Fig. 1b). The LysR family transcriptional regulator TtdR (F6476_RS01225) is located downstream of F6476_RS01220. F6476_RS05835 is located 918 kb away from F6476_RS01220 and is co-localized with the genes that encode enzymes catalyzing the conversion of glyoxylate to pyruvate. A TetR/AcrR family transcriptional regulator is located close to this operon. Considering the presence of all EG oxidation complements downstream of F6476_RS01220, TtdR became the focus of this study.

**Fig. 2:**
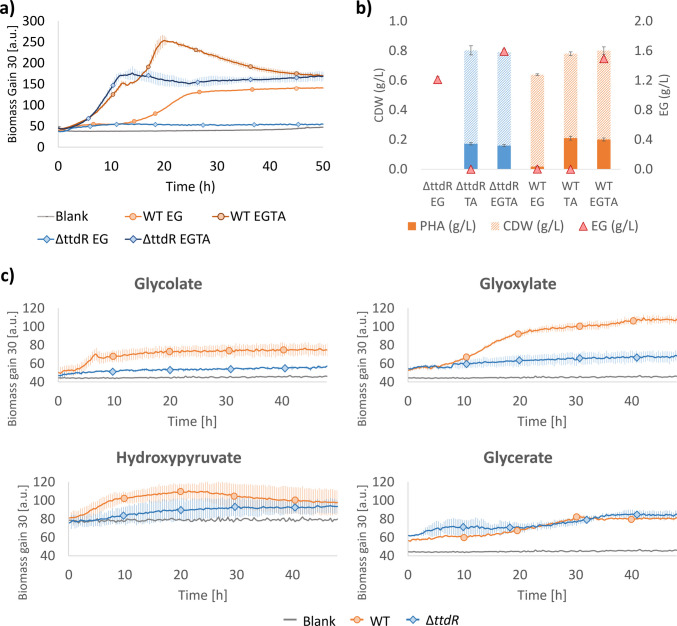
Growth and PHA production of GO16 WT (orange color, circle) and GO16 Δ*ttdR* (blue color, diamond). **a** Growth of *P. umsongensis* GO16 WT vs Δ*ttdR* in MSM_full_ (1 g of NH_4_Cl L^−1^) with EG (30 mM, 1.86 g L^−1^) as a sole carbon and energy source, or when EG (30 mM) and TA (20 mM, 4.2 g L^−1^) were used as co-substrates in a BioLector (Flowerplate MTP 48-well, BioLector® I, BECKMAN COULTER Life Sciences). Blank is a non-inoculated culture medium. Data represents the mean of three biological replicates, each analyzed in technical triplicate (*n* = 9 total measurements); error bars represent the standard deviation (SD). **b** Biomass as cell dried weight (CDW), PHA accumulation and EG consumption in 50 mL MSM_lim_ (0.25 g of NH_4_Cl L.^−1^) after 48 h cultivation. Data points represent the mean of three independent biological replicates (*n* = 3); error bars represent the standard deviation (SD). **c** Growth of GO16 WT and GO16 Δ*ttdR* strains in a BioLector using EG oxidation intermediates: glycolate, glyoxylate, hydroxypyruvate, and glycerate. The strains were cultured overnight in 10 mL of MSM_full_ with 20 mM TA. Then, bacterial cultures with a starting OD_540nm_ of 0.1 were inoculated into MSM_full_ containing 10 mM of glycolate, glyoxylate, hydroxypyruvate, or glycerate. The cells were cultivated at 30 °C and 1000 rpm for 48 h. The 10 mM hydroxypyruvate solution possesses a light brown color, which results in a higher baseline absorbance compared to the other, colorless intermediates. All experiments were conducted in biological triplicates. Blank: uninoculated medium. (*n* = 9)

To investigate if TtdR has a role in EG metabolism, the *ttdR* gene was deleted. *P. umsongensis* GO16 Δ*ttdR* was cultivated in a BioLector with 30 mM EG (1.86 g L^−1^) as the sole carbon and energy source, or a mixture of 20 mM TA (4.2 g L^−1^) and 30 mM EG as co-substrates in MSM_full_ (Fig. 2a). GO16 Δ*ttdR* failed to grow when EG served as the sole carbon and energy source. The GO16 WT exhibited a prolonged lag phase but ultimately grew on EG as the sole carbon and energy source.

When a mix of TA and EG was supplied, a similar growth pattern was observed for both strains in the first 10 h, likely due to the preferential use of TA (Tiso et al. 2021). While the WT continued to accumulate biomass after this period, the GO16 Δ*ttdR* strain entered the stationary phase (Fig. 2a).

This initial analysis was followed by 50 mL liquid culture growth experiments. Again, no biomass was detected when GO16 Δ*ttdR* was cultivated with EG as the sole carbon and energy source (Fig. 2b). To test the viability of the strain, GO16 Δ*ttdR* was also cultivated with TA only, and the biomass obtained was comparable to the WT (Fig. 2b), confirming the effect was specific to EG metabolism. It was previously described that in the presence of the GclR repressor, *P. putida* KT2440 could use EG only as an energy source (Li et al. 2019). When EG was solely supplied, 35% (0.66 ± 0.03 g L^−1^) of EG was depleted from the GO16 Δ*ttdR* supernatant under both non-limiting and limiting conditions with little to no biomass, while in the case of mixed TA and EG only 10% of EG was consumed within 48 h of cultivation under limiting condition (Fig. 2b). Under the same conditions, the WT completely consumed EG, yielding 0.62 ± 0.005 g L^−1^. It is worth mentioning that the WT only consumed 20% (0.37 ± 0.02 g L^−1^) of EG when supplied in mix with TA under nitrogen-limiting conditions, whereas the WT was able to consume all of the EG under nitrogen-full conditions.

After it was confirmed that the deletion strain couldn’t grow on EG as a sole carbon and energy source, the next step was to investigate which part of the EG metabolism was affected. When 10 mM of EG oxidation intermediates were used, GO16 Δ*ttdR* showed no growth on glycolate or glyoxylate, and exhibited poor growth on hydroxypyruvate, while the GO16 WT grew on all three substrates (Fig. 2c). Both the GO16 Δ*ttdR* and the WT grew similarly on glycerate, indicating that TtdR is specifically involved in the conversion from glycolate to glycerate.

**Fig. 3:**
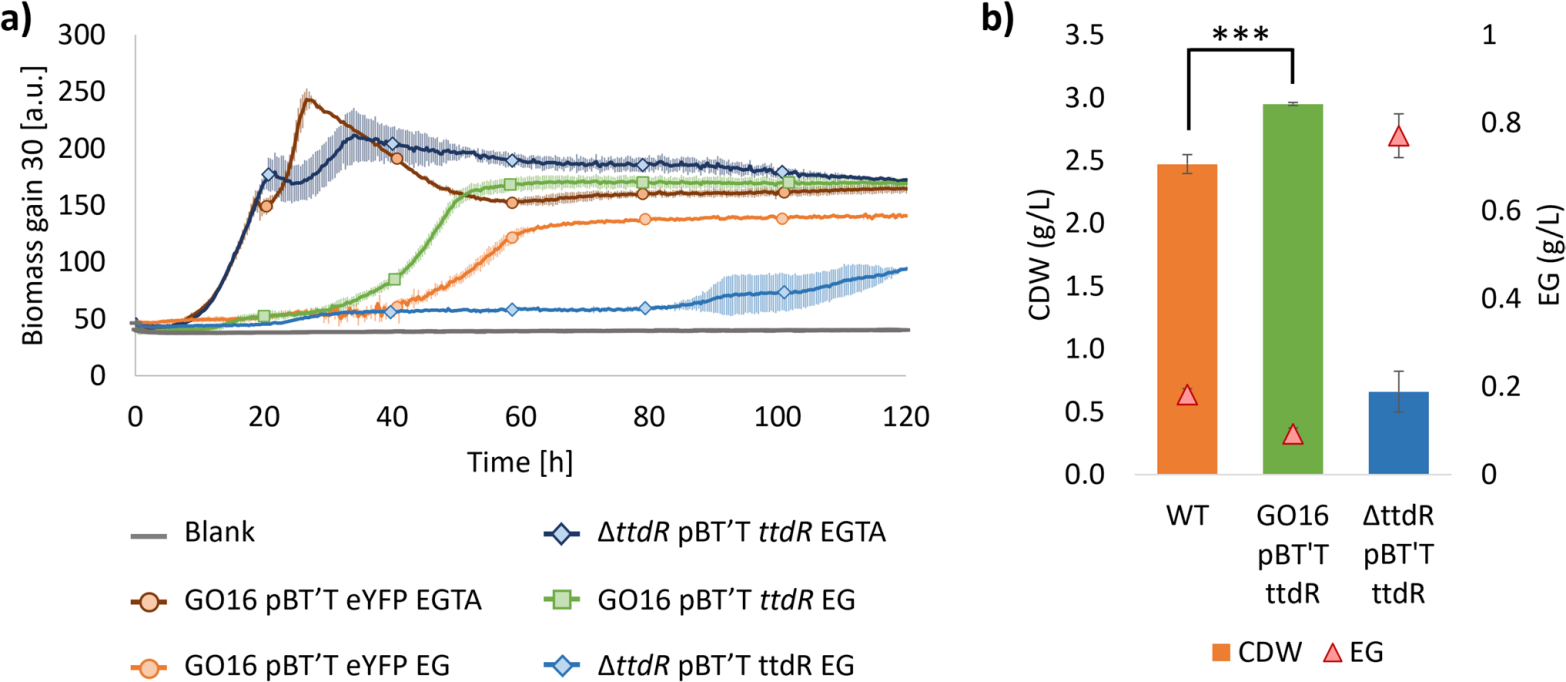
Growth of *P. umsongensis* GO16 WT (Orange), GO16 pBT′T eYFP (Circle), GO16 pBT′T_*ttdR* (green, square), and Δ*ttdR* complemented with pBT′T_*ttdR* (Δ*ttdR* pBT′T_ttdR) (blue, diamond). **a** Cultivation on MSM_full_ supplemented with 30 mM EG, or 20 mM TA and 30 mM EG (EGTA) in a BioLector. (*n* = 9). **b** Flask culture in 50 mL MSM_full_ with 120 mM EG at 30 °C, 200 rpm for 48 h (*n* = 3). A two-sample *t*-test was performed for GO16 WT and GO16 pBT′T_*ttdR* samples cultivated on 120 mM EG (*t* = − 10.79, *p* = 0.00042), showing a statistically significant difference between the groups (*p* < 0.0005)

TtdR is annotated as a LysR-type transcriptional regulator, which is mostly known as an activator in *Pseudomonas* species (Maddocks and Oyston 2008). This is in agreement with the lack of growth of GO16 Δ*ttdR* on EG, glycolate, or glyoxylate. Thus, it is likely that TtdR is an activator for EG metabolism in GO16, and complementation of GO16 Δ*ttdR* should result in growth with EG. The *ttdR* gene was expressed using pBT′T plasmid which possesses a constitutive P_*tac*_ promoter (Koopman et al. 2010). When grown with EG as a sole carbon and energy substrate, GO16 Δ*ttdR* pBT′T_*ttdR* recovered its ability to utilize EG; however, a long lag phase was observed (light-blue line; Fig. 3a). Also, when TA and EG were co-fed in GO16 Δ*ttdR* pBT′T_*ttdR*, a characteristic two-phase growth was observed, suggesting preferential consumption of TA, followed by EG utilization (dark-blue line). While the two-phase growth was also observed when TtdR was overexpressed in the GO16 WT (green line), this strain displayed a 10-h shorter lag phase and 1.25-fold higher biomass compared to the control strain, GO16 pBT′T_eYFP (orange line) with EG as sole carbon source.

The strains were also grown in flasks containing 50 mL MSM_full_ supplemented with 120 mM EG (7.45 g L^−1^) as a sole carbon source (Fig. 3b). GO16 Δ*ttdR* pBT′T_*ttdR* consumed 6.68 g L^−1^ of EG and accumulated 0.66 ± 0.16 g L^−1^ of CDW, which confirms that intrans expression of TtdR restores the growth of GO16 Δ*ttdR* with EG. When TtdR was overexpressed in the WT, only 0.09 ± 0.01 g L^−1^ of EG left after 48 h and 1.2-fold higher CDW was achieved. Again, these results show that TtdR is a key activator of EG metabolism in GO16.

### Growth and PHA accumulation by GO16 Δ*ttdR* and other carbon sources

**Fig. 4:**
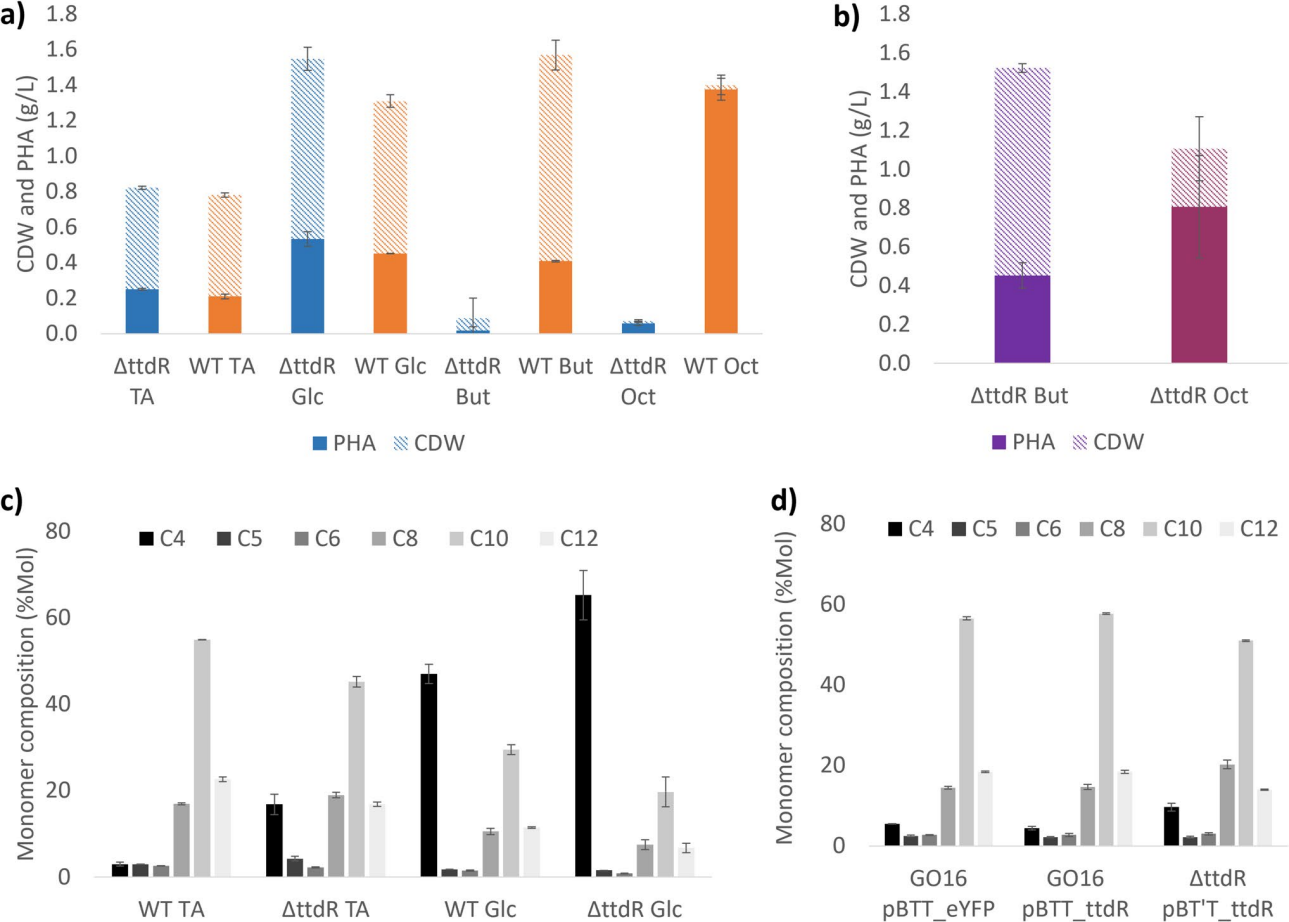
Biomass and PHA accumulation when cultivated on the other carbon substrates: terephthalic acid (TA), glucose (Glc), butyrate (But), or octanoate (Oct). **a** Flask cultivation of Δ*ttdR* (Blue) and GO16 WT (Orange) for 48 h in 50 mL MSM_lim_ (*n* = 3). The carbon and energy substrates were supplied to correspond to 1.96 g_c_ L^−1^. **b** Five-day flask cultivation of GO16 Δ*ttdR* with butyrate (But, 40 mM, violet) or octanoate (Oct, 20 mM, Purple) in 50 mL MSM_lim_. **c** PHA monomers of GO16 WT and Δ*ttdR* produced after 48 h grown on 20 mM TA or 26.5 mM glucose in MSM_lim_. **d** PHA monomer composition of GO16 pBT′T_eYFP, GO16 pBT′T_*ttdR*, and GO16 Δ*ttdR* pBT′T_*ttdR*. Strains were harvested after 48 h grown on 20 mM TA in MSM_lim._ All experiments were conducted in biological triplicates

As EG belongs to the so-called PHA carbon-unrelated substrates, along with TA and glucose, it is important to impose an inorganic nutrient limitation, such as nitrogen limitation, to support PHA accumulation (Ward et al. 2005). GO16 Δ*ttdR* showed similar biomass and PHA accumulation to the WT when TA or glucose were used as a sole carbon source, suggesting that *ttdR* deletion is not negatively affecting TA or glucose metabolism (Fig. 4a). Fatty acids, such as butyrate or octanoate have been shown to support both biomass and PHA accumulation by GO16 WT (Cerrone et al. 2023). However, when GO16 Δ*ttdR* was cultivated with 40 mM butyrate or 20 mM octanoate, a negligible biomass was observed after 48 h of cultivation (Fig. 4a). GO16 Δ*ttdR* started to grow after 3 days when either 40 mM butyrate or 20 mM octanoate were used. After 5 days of cultivation, GO16 Δ*ttdR* achieved 1.52 ± 0.11 g L^−1^ of CDW with butyrate, which is comparable to the growth of WT, and 1.11 ± 0.16 g L^−1^ of CDW with octanoate, a 1.2-fold lower biomass compared to the WT (Fig. 4b). This prolonged lag phase suggested a link between the TtdR regulator and fatty acid metabolism. While biomass and PHA level did not differ between GO16 Δ*ttdR* and the WT strains when grown with glucose or TA in PHA accumulating conditions, i.e., MSM_lim_, a difference was observed in terms of PHA monomer composition (Fig. 4c).

As previously stated, GO16 contains both scl and mcl-PHA synthesis pathways (Cerrone et al. 2023; Narancic et al. 2021), and accumulates two distinct polymers, scl-PHAs, composed of (*R*)−3-hydroxybutyrate (C4) and (*R*)−3-hydroxyvalerate (C5), and mcl-PHAs composed of (*R*)−3-hydroxyhexanoate (C6), (*R*)−3-hydroxyoctanoate (C8), (*R*)−3-hydroxydecanoate (C10), and (*R*)−3-hydroxydodecanoate (C12). GO16 Δ*ttdR* consistently showed increased accumulation of C4 monomer across all carbon sources tested, accompanied by a corresponding reduction in the C10 and C12 fractions in the accumulated mcl-PHA. Under TA conditions, GO16 Δ*ttdR* accumulated 20 mol% C4, a significant increase compared to only 3 mol% in the WT (Fig. 4c). A similar trend was observed with glucose, where Δ*ttdR* accumulated 65 mol% C4 monomers, compared to 47 mol% in the WT. Conversely, overexpression of TtdR in the GO16 WT background reduced C4 monomers. When TA was used, this strain showed a 1.7-fold decrease in the proportion of the C4 monomer compared to the GO16 pBT′T_eYFP control (Fig. 4d). Collectively, these results strongly suggest that the absence of TtdR somehow promotes scl-PHA accumulation, overriding the typical monomer distribution regardless of the substrate used.

### Proteomic analysis reveals proteins regulated by TtdR

**Fig. 5:**
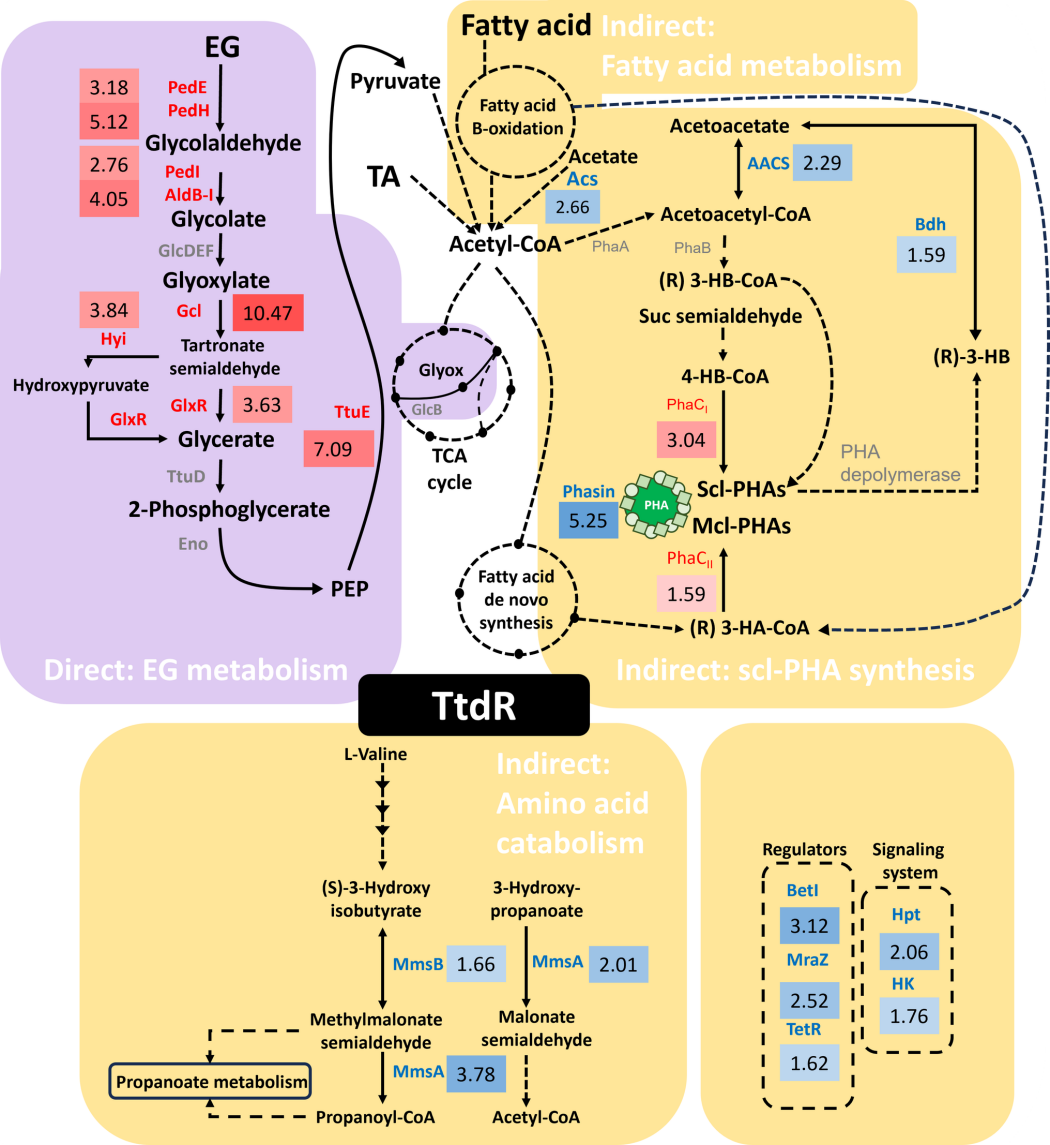
The direct and indirect regulation of TtdR revealed by proteomic changes in *P. umsongensis* GO16 Δ*ttdR*. Direct regulation (EG metabolism) is colored as light-purple and indirect regulations are colored as light-yellow. Proteins more abundant in the ∆*ttdR* are shown in blue, while those more abundant in the WT are colored red. Reactions catalyzed by these proteins are indicated by solid lines, while all others are dashed. Numbers next to each protein indicate the log₂ fold change (logFC) observed in the proteomics data. A logFC of +1 or higher indicates the protein is at least twofold more abundant in WT than in Δ*ttdR*, whereas a logFC of −1 or lower indicates the protein is at least twofold more abundant in Δ*ttdR* than in WT. Acs, acetyl-CoA synthetase; AACS, acetoacetyl-CoA synthase; Bdh, 3-hydroxybutyrate dehydrogenase; MmsB, 3-hydroxyisobutyrate dehydrogenase; MmsA, (methyl)malonate-semialdehyde dehydrogenase; Hpt, Hpt-domain containing response regulator; HKs, histidine kinase; PhaA, β-ketothiolase; PhaB, acetoacetyl-CoA reductase; PhaC, PHA synthetase; PedEH, quinoprotein alcohol dehydrogenases; PedI and AldB-I, aldehyde dehydrogenases; GlcDEF, glycolate oxidase; Gcl, glyoxylate carboligase; Hyi, hydroxypyruvate isomerase; GlxR, tartronate semialdehyde reductase; TtuD, hydroxypyruvate reductase; Eno, phosphopyruvate hydratase; TtuE, pyruvate kinase; AceA, isocitrate lyase; GlcB, malate synthase; HA, hydroxyacyl; HB, hydroxybutyrate

TtdR has a wider effect than just activating EG catabolism in GO16. To try and understand how TtdR and PHA accumulation are connected, a shotgun proteome analysis was carried out. GO16 WT and GO16 Δ*ttdR* were cultivated under PHA non-accumulating, i.e., MSM_full_, or PHA accumulating, i.e., MSM_lim_, conditions. As GO16 Δ*ttdR* canot grow with EG only, a mix of 20 mM TA and 30 mM EG was used (Fig. 5 and Fig. S2 and Tables S3–S4). As a control, the strains were grown on 20 mM TA or 26.5 mM glucose under PHA accumulating and non-accumulating conditions and collected at 48 h (Tables S5–S8).

EG-related enzymes, PedH, PedE, PedI, Gcl, TtuE, Hyi, and GlxR were more abundant in GO16 WT compared to GO16 Δ*ttdR* when TA/EG mix was used as feedstock. In addition, both type I and II PhaCs were more abundant in the GO16 WT at 24 h under PHA non-accumulating conditions when grown on a mixture of TA and EG, even though no PHA was detected in the samples. Phasins were fivefold more abundant in the GO16 Δ*ttdR* under the same conditions.

Among the proteins more abundant in the GO16 Δ*ttdR*, 3-hydroxybutyrate dehydrogenase (Bdh; F6476_RS11380; EC1.1.1.30) was identified when either EG and TA or glucose were used under PHA-accumulating conditions (Tables S4, S6, and S8). Acetoacetyl-CoA synthase (AACS; F6476_RS11375) was twofold more abundant in the GO16 ∆*ttdR* when grown on glucose under PHA-accumulating conditions (Table S8). This protein did not show statistical significance when the strains were cultivated on TA or on TA and EG. A 3-hydroxyisobutyrate dehydrogenase (MmsB; F6476_RS00545; EC1.1.1.31) and a (methyl)malonate-semialdehyde dehydrogenase (MmsA; EC1.2.1.27), both annotated as enzymes involved in L-valine degradation (Marshall and Sokatch, 1972), showed higher abundance in GO16 Δ*ttdR*, regardless of substrate used (Tables S4-S8). There are two copies of *mmsA* in the chromosome of GO16 (F6476_RS00540 and F6476_RS08430), and both enzymes were more abundant in GO16 Δ*ttdR* grown with EG and TA under PHA-accumulating conditions. Moreover, acetyl-CoA synthetase (Acs; F6476_RS08435; EC 6.2.1.1) was 2.6-fold more abundant under PHA-accumulating conditions in GO16 Δ*ttdR* when grown on EG and TA. Acs catalyzes the formation of acetyl-CoA from acetate and coenzyme A, potentially increasing the acetyl-CoA pool available for PHB synthesis.

### TtdR–fatty acids link through adapted laboratory evolution (ALE)

When fatty acids are used as carbon and energy substrates during aerobic growth, acetyl-CoA is produced, and if sent to the TCA cycle, two carbon dioxide molecules would be released leading to no net production of biomass (Clark and Cronan 2005). Therefore, the glyoxylate shunt is used to avoid the depletion of TCA cycle intermediates. Since GO16 exhibited impaired growth with fatty acids, and due to the overlap in the reactions of EG catabolism and glyoxylate shunt, GO16 Δ*ttdR* was evolved through ALE for growth with butyrate or octanoate. These two fatty acids were chosen because of their different chain lengths, and therefore different numbers of β-oxidation cycles required and concomitantly different metabolic flux distribution through the glyoxylate shunt. Furthermore, butyrate would support accumulation of scl-PHA i.e., polyhydroxybutyrate, while octanoate leads to mcl-PHA accumulation with mainly C8 monomer. It was hypothesized that the mutations acquired during the ALE may reveal insights into the regulatory mechanism of TtdR and complement the analysis of the proteome.

**Fig. 6:**
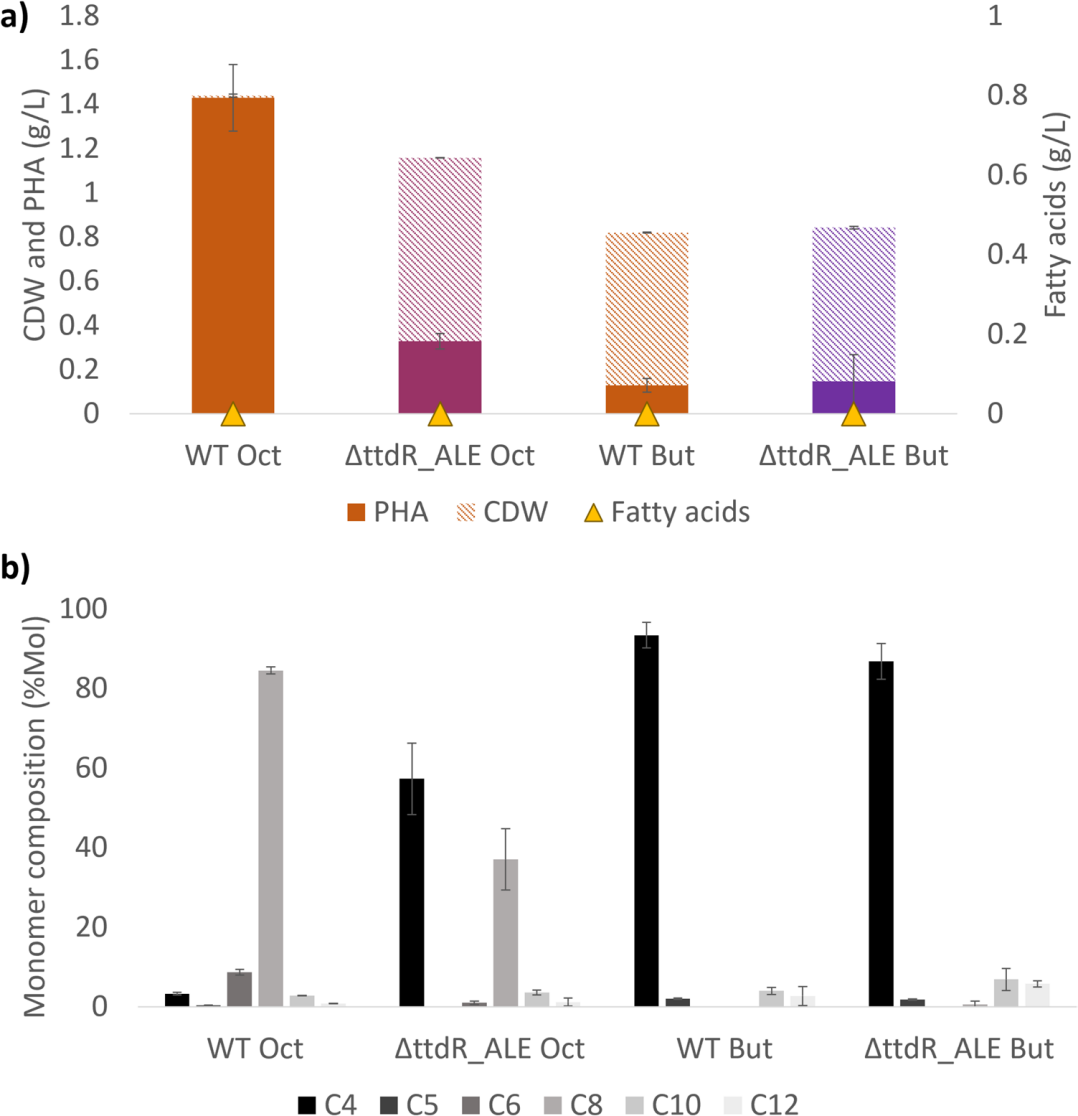
Biomass and PHA accumulation of ALE strains on fatty acids. **a** Cell dried weight (CDW) and PHA produced during the cultivation of GO16 WT (orange), Δ*ttdR*_ALE_Oct_ (magenta), and Δ*ttdR*_ALE_But_ (violet) strains in 250 mL flasks containing MSM_lim_ 20 mM octanoate (Oct) or 20 mM butyrate (But) at 30 °C and 200 rpm for 48 h. **b** PHA monomer composition. All experiments were conducted in biological triplicates

As shown in Fig. 4b, only when cultivation was extended to 5 days did GO16 Δ*ttdR* achieve biomass comparable to the WT cultivated for 48 h with butyrate and 1.26-fold lower biomass with octanoate. Three colonies isolated from these cultures were used in the ALE. The 4th passage achieved OD_540nm_ = 13 from octanoate and OD_540nm_ = 7 from butyrate in 48 h cultivation respectively, and was used to obtain single colonies. Three colonies of each Δ*ttdR*_ALE_Oct_ and Δ*ttdR*_ALE_But_ were cultured in the flasks, and they appeared to have recovered the growth with butyrate or octanoate when MSM_full_ was used (Fig. S3). However, under PHA accumulating conditions, Δ*ttdR*_ALE_Oct_ accumulated 1.24-fold lower biomass compared to WT grown under the same conditions (Fig. 6a). This was largely due to the 4.4-fold decrease in accumulated PHA, suggesting that PHA metabolism was affected. Δ*ttdR*_ALE_Oct_ also showed a higher proportion of scl-PHA, 57 mol% of (*R*)−3-hydroxybutyrate compared to the WT that had only 3 mol% of this monomer (Fig. 6b). Δ*ttdR*_ALE_But_ strain showed a very similar biomass, PHA level and monomer profile to the GO16 WT strain when grown with butyrate.

While the growth with fatty acids was restored, neither of the ALE strains could use EG as a carbon substrate, nor acetate (Fig. S4), also assimilated via glyoxylate shunt (Dolan and Welch 2018; Kornberg and Quayle 1958).

The three individual strains evolved for each condition were sequenced and the genomes were compared to the GO16 Δ*ttdR*. Multiple single nucleotide polymorphisms (SNPs) were identified across strains (Table 2). Mutations shared by both the ALE_But_ and ALE_Oct_ strains included those in genes encoding an aldehyde dehydrogenase (F6476_RS27365), a PQQ-dependent dehydrogenase (F6476_RS12110), and a folate synthase FolK (F6476_RS28160). Condition-specific mutations were also observed: *uvrY* (F6476_RS11445) for the ALE_But,_ while the medium-chain-length PHA synthase gene, *phaC* (F6476_RS32376), was modified in the ALE_Oct_ strains. In the *phaC*, a cytosine deletion at position 480 bp resulted in a frameshift mutation, and a glycine at CDS position 1432 was substituted with tryptophan (G478W). Due to the frameshift, nucleotides at positions 565–567 were translated as a stop codon (amino acid position 189), causing premature termination. These mutations are the reason for the significant drop in PHA accumulation in the ALE_Oct_ strains compared with the GO16 WT strain and increase in C4 fraction when octanoate was used (Fig. 6b).

**Table 2:** List of genes affected in the evolved ALE_But_ and ALE_Oct_ strains and the corresponding changes

Strain	Affected locus	Pathway/COG	Function/annotation	Mutation	AA substitution
ALE_But_, ALE_Oct_	RS12110	Methanol/ Alcohol oxidation	PQQ-dependent dehydrogenase	409 G—> A	E137K
ALE_But_	RS27365	Intermediate metabolism/detoxification	Aldehyde dehydrogenase family protein	365 CC—> TG 383 T—> A	A122V F128Y
ALE_Oct_				307 T—> A 365 CC—> TG 383 T—> A 415 GCT—> TCG	S103T A122V F128Y A139S
ALE_But_, ALE_Oct_	RS28160	Folate biosynthesis	Folate synthesis protein (FolK)	267 C—> A	L89F
ALE_But_	RS11445	Global regulation of carbon metabolism	Response regulator (UvrY)	488 G—> T	C163F
ALE_Oct_	RS32375	PHA metabolism	mcl-PHA synthase (PhaC)	1432 C—> A 480 -C	G478W Early termination

## Discussion

TtdR is the activator of EG metabolism in *P. umsongensis* GO16, suggesting a different regulation mechanism compared to the EG metabolism in KT2440 (Li et al. 2019) and of JM37 (Li et al. 2010). TtdR functions as a pleiotropic regulator that coordinates the primary utilization of EG with broader cellular metabolic flux. Our data suggest that TtdR exerts its influence across four metabolic axes. Most prominently, TtdR directly activates the glycolate–glyoxylate pathway (Figs. 1 and 2). This primary activation triggers a series of indirect effects, including the stimulation of fatty acid metabolism via the glyoxylate shunt and the modulation of the acetyl-CoA pool, which directly impacts the synthesis of scl-PHA. Furthermore, TtdR indirectly promotes amino acid catabolism through the MmsA/MmsB pathway. This multi-layered regulatory network, illustrated in Fig. 5, demonstrates how TtdR synchronizes carbon source utilization with secondary metabolism and energy storage.

The deletion of TtdR could disrupt the complex regulatory networks in which it participates, leading to altered expression of other regulators, including LTTRs and their target genes. A number of LTTRs were located near genes encoding proteins that showed differential abundance in the GO16 Δ*ttdR* strain, suggesting a potential link (Fig. S5). Therefore, some proteins that do not have a direct link with EG metabolism, glyoxylate shunt, or PHA synthesis were also detected in the proteomics data.

The link between fatty acid and EG metabolism is presented by the reactions of the glyoxylate shunt, which converts acetyl-CoA into four-carbon dicarboxylic acids (malate and succinate) without the loss of carbon atoms as CO_2_, especially when acetate or fatty acids are the main carbon and energy sources (Clark and Cronan 2005; Fujita et al. 2007; Maloy et al. 1980; Rabin et al. 1965). The key enzymes of glyoxylate shunt are malate synthase GlcB, along with isocitrate lyase AceA (Franden et al. 2018; Li et al. 2010). The expression of these two genes is significantly increased when exposed to various fatty acids, including butyrate and octanoate (Thompson et al. 2020). Therefore, it is not surprising that the absence of TtdR led to a 3-day lag phase when GO16 Δ*ttdR* was cultivated with fatty acids. While the ALE process employed in this study led to the growth of the evolved strains with fatty acids, these strains still weren’t able to grow with EG or acetate. While it is difficult to gauge this from the mutations found in the ALE strains, it could be that the evolved strains may have adapted by rerouting acetyl-CoA from even-numbered fatty acids into alternative pathways. Some species that lack a complete glyoxylate shunt, such as *Rhodospirillum rubrum*, *Rhodobacter sphaeroides*, and *Methylobacterium extorquens*, employ different metabolic strategies to assimilate C2 compounds like acetate and even-chain fatty acids (Alber et al. 2006; Leroy et al. 2015; Narancic et al. 2016; Schneider et al. 2012; Shimizu et al. 2022). These bacteria rely on an ethylmalonyl-CoA (EMC) pathway, which shares the reactions with the PHB synthesis pathway, namely the condensation of two acetyl-CoA to acetoacetyl-CoA, and reduction to 3-hydroxybutyryl-CoA. Further conversion to succinate requires EMC core enzymes crotonyl-CoA carboxylase (Ccr) and ethylmalonyl-CoA epimerase (Epi), which are not present in GO16. However, it could be that there is an increased flux through PHB synthesis in the absence of glyoxylate shunt, leading to higher C4 fraction. *R. sphaeroides* and *R. rubrum* both exhibited impaired growth with acetate when PHA synthesis was disabled via *phaC1 *or* phaC1 *and* phaC2* deletion (Narancic et al. 2016; Shimizu et al. 2022).

The question that remains then is how the TCA cycle intermediates are replenished when fatty acids are used as a substrate, and the glyoxylate shunt is disabled. Both ALE strains obtained a missense mutation in FolK (L89F), which is a key enzyme in the biosynthesis of tetrahydrofolate (THF). Structural modeling via AlphaFold indicates that the L89F substitution, replacing a flexible leucine with a bulky phenylalanine, occurs near the catalytic loop. This mutation likely alters the enzyme’s gating mechanism or relieves feedback inhibition by the product HMDP-PP (6-hydroxymethyl-7,8-dihydropterin pyrophosphate), thereby increasing the cellular pool of THF. An increased THF supply is critical because it serves as the mandatory C1 acceptor for the Glycine Cleavage System (GCS). Parallel to the FolK mutation, synonymous SNPs in two distinct GcvP loci (RS19155 and RS24260) were identified. One possible compensatory route can be GCS-Serine bypass. In this model, glycine (likely derived from glyoxylate or amino acid pools) is decarboxylated by the GCS to generate one carbon 5,10-methylene-THF (Lehninger 2004; Moreno et al. 2009). This C1 unit is then assimilated via the serine cycle to produce pyruvate. Pyruvate can then be converted to oxaloacetate for TCA replenishment or utilized in gluconeogenesis (Bruinsma et al. 2023). One thing to note is that this pathway is energetically demanding. The growth on butyrate or octanoate but not acetate may be explained by the high yield of NADH and FADH_2_ from fatty acid *β*-oxidation, which sustain this less efficient C1 dependent bypass. Further work is needed to investigate the role of GCS-Serine bypass, but this integrated pathway may offer a conversion of fatty acid-derived carbon into biomass precursors, circumventing the need for the glyoxylate shunt.

When it comes to the link of TtdR and PHA monomer composition, this could be potentially explained by looking at the differential abundance of proteins in GO16 Δ*ttdR* and WT grown under PHA accumulating conditions (Table S4, S6, S8). GO16 contains both scl and mcl-PHA synthesis pathways, and the two discrete polymers are blended on the PHA granule level (Cerrone et al. 2023). The majority of substrates, including octanoate and TA, give rise to predominantly mcl-PHA in GO16, while butyrate yields predominantly scl-PHA (> 95% C4). While GO16 Δ*ttdR* showed similar biomass as the WT when TA or glucose were used as substrates, and similar PHA level (%CDW), the monomer composition was different. Under PHA-accumulating conditions, GO16 Δ*ttdR* showed increased C4 monomer by 6.6-fold when TA was a sole substrate, while mcl-PHA monomers were decreased, leaving the overall PHA level similar to the WT (Fig. 4). Increased abundance of Bdh, MmsA, and MmsB in GO16 Δ*ttdR* combined could contribute to increased C4 monomer synthesis (Fig. 5/Table S6). While Bdh traditionally oxidizes (*R*)−3-hydroxybutyrate into acetoacetate for metabolism, it may also catalyze the reverse reaction to increase the C4 PHA monomer fraction (Aneja and Charles 1999; Machado et al. 2020). This dual role suggests that instead of solely aiding degradation, the enzyme could actively contribute to the production of the scl-PHA in GO16 Δ*ttdR*. AACS, which was abundant in the GO16 ∆*ttdR* when cultured on glucose under PHA-accumulating conditions, also remains of interest because the *aacs* gene, which is located downstream (3′) of bdh on the GO16 chromosome, catalyzes the conversion of acetoacetate to acetyl-CoA, which subsequently proceeds to 3-HB-CoA, a precursor of scl-PHA.

Additionally, the MmsAB system, which typically converts 3-hydroxyisobutyrate to propionyl-CoA, exhibited increased abundance in Δ*ttdR* (Chowdhury et al. 1996; Hanko et al. 2017). Propionyl-CoA can be converted into succinate, a TCA cycle intermediate, via the 2-methylcitrate cycle (2MCC) (Dolan et al. 2022). However, we did not observe any differences in 2MCC protein levels in the proteomics data. MmsB and MmsA enzymes possess broad substrate specificity (Li et al. 2020). The accumulating glycolate or glyoxylate might act as a structural mimic of the hydroxybutyrate/propanoate intermediates and their semialdehydes, which these enzymes typically process to eliminate toxic accumulations. Additionally, when MmsA utilizes malonate semialdehyde as a substrate, acetyl-CoA, NADH, and CO_2_ are produced (Talfournier et al. 2011). Propionyl-CoA and acetyl-CoA are both precursors for scl-PHA synthesis (Borrero‐de Acuña and Poblete‐Castro 2023; Catalán et al. 2018). While (*R*)−3-hydroxyvalerate (C5) amounts showed no difference, the higher abundance of MmsA and MmsB may be contributing to the increased C4 content. In fact, it was shown that the *mmsA* deficient *Rhodobacter capsulatus* had reduced PHB synthesis (Zhang et al. 2016).

Based on these results, one can envisage that GO16 Δ*ttdR* could be exploited further to control the ratio of scl and mcl-PHA in a substrate-independent manner, with the aim of improving the material properties of the resulting material. In GO16, mcl- and scl-PHA are produced as individual polymers and blended in vivo, with mcl-PHA acting as a plasticizer, improving the properties of otherwise brittle PHB (Bhola et al. 2021; Cerrone et al. 2023). The future work may explore how TtdR expression level could be manipulated to control the scl-PHA content regardless of the substrate used.

## Conclusions

In this work we have found that a LysR-type transcriptional regulator TtdR exerts control over multiple pathways in *P. umsongensis* GO16. *TtdR* is located at 5′ of the gene cluster involved in EG metabolism and is acting as a crucial activator of the pathway. The absence of *ttdR* renders the GO16 mutant incapable of utilizing EG as its sole carbon and energy source. While this work shows the link of TtdR, fatty acids metabolism, and other auxiliary pathways, such as amino acid catabolism, from the applicative side the effect on PHA metabolism may be the most interesting. GO16 Δ*ttdR* accumulated more scl-PHA than mcl-PHA regardless of the carbon source used. Considering the capacity of GO16 to blend scl- and mcl-PHA, regulating the amount of each polymer in a carbon feedstock non-specific manner would offer an advantage in the production of PHA blends.

## Supplementary Information

Below is the link to the electronic supplementary material.

ESM1 (DOCX 572 KB)

## Data Availability

The proteomics datasets generated and analyzed in this study were deposited in the PRIDE partner repository of the ProteomeXchange Consortium and are publicly available under the accession numbers PXD075284 and PXD075327.
