# Supplementary material for: LysR-type transcriptional regulator TtdR regulates both ethylene glycol and polyhydroxyalkanoate (PHA) metabolism in Pseudomonas umsongensis GO16
Source: Appl Microbiol Biotechnol. 2026 May 19;110:211. doi: 10.1007/s00253-026-13865-3 (PMC13357446; doi:10.1007/s00253-026-13865-3)
Supplement: Supplementary file 1 [file 253_2026_13865_MOESM1_ESM.docx]

**Supplementary data for**

LysR type transcriptional regulator TtdR regulates both ethylene glycol and polyhydroxyalkanoate (PHA) metabolism in *Pseudomonas umsongensis* GO16

Jounghyun Um^1^, Karthika Balusamy^1^, Nick Wierckx^3^, Kevin E. O’Connor^1,2^, Tanja Narancic^1,2*^

^1^UCD Earth Institute and School of Biomolecular and Biomedical Science, University College Dublin, Belfield, Dublin 4, Ireland

^2^BiOrbic - Bioeconomy Research centre, Ireland, University College Dublin, Belfield, Dublin 4, Ireland

^3^Institute of bio- and Geosciences IBG-1: Biotechnology, Forschungszentrum Jülich, Jülich, Germany

*Corresponding author: Dr Tanja Narancic, BiOrbic and School of Biomolecular and Biomedical Sciences, Earth Institute, O’Brien Centre for Science, University College Dublin, Belfield, Dublin 4, Ireland Telephone: +353 1 716 2679; E-mail: [tanja.narancic@ucd.ie](mailto:tanja.narancic@ucd.ie)

CDW / PHA [g/L]

TA / EG [mM]

|  | PHA [g/L] | CDW - PHA | TA [mM] | EG [mM] | GLU [mM] |
| --- | --- | --- | --- | --- | --- |
| WT TA 48hr | 0.237653 | 0.60368 | 0 |  |  |
| ΔgclR TA 48hr | 0.0979869 | 0.597346 | 0.00861417 |  |  |
| WT EG 48hr | 0.150024 | 0.997309 |  | 2.69736 |  |
| ΔgclR EG 48hr | 0.0045352 | 0.559465 |  | 29.628 |  |
| WT TA+EG 48hr | 0.175697 | 0.954303 | 0 | 0 |  |
| ΔgclR TA+EG 48hr | 0.149838 | 0.806828 | 0 | 0 |  |
| WT GLU 48hr | 0.447524 | 0.805143 |  |  | 0 |
| ΔgclR GLU 48hr | 0.272579 | 0.902087 |  |  | 0 |

**Figure S1** F6476_RS21810 deletion: Cell dry weight, PHA content, and carbon consumption in P. umsongensis WT and F6476_RS21810 knockout mutant (ΔgclR) at 48 h of cultivation. Growth assays were carried out in 50 mL Nitrogen limited Minimal salts medium (MSM_lim_), containing 1.96 g L^-1^ of Carbon (C) source (20 mM TA, 82 mM EG, 27 mM glucose and in case of mixed C sources, the 1.96 g L^-1^ of C was contributed equally by different C sources). Experiment conducted by K. Balusamy.

**Figure S2** Proteomics analysis of GO16 vs ttdR knockout. The strains were cultured using 250 mL flasks with 30 mM EG and 20 mM TA in MSM nitrogen full or limited condition. Samples were collected at 24 hours and 48 hours. Three biological replicates per sample were used for proteomics analysis. A) The overlap of identified proteins between samples in interactive heatmaps. B) Volcano plots which show differentially abundant proteins in GO16 WT vs ΔttdR cultured for 48 hours in nitrogen-full condition. Proteins significantly abundant in the WT are marked in pink, and the one in ΔttdR is denoted in light blue.

**Figure S3** Adaptive laboratory evolution of P. umsongensis GO16 ΔttdR on (A) 20 mM octanoate and (B) 20 mM butyrate in 250 mL flasks containing MSM_full_. Three parallel flask cultivations were carried out and OD_540nm_ was measured after 48-72 h of cultivation.

|  | CDW (g/L) |
| --- | --- |
| GO16 | 1.23933 |
| ΔttdR | 0.024 |
| ΔttdR_ALE Oct | 0.165 |
| ΔttdR_ALE But | 0.019 |

**Figure S4** Growth test on 80 mM acetate in MSM_full_. GO16 WT was harvested after 48 h, and other strains (ΔttdR, ΔttdR_ALE_Oct_, and ΔttdR_ALE_But_) were collected after 5 days.

**Figure S5** Location of significant proteins and LysR-type transcriptional regulators (LTTRs) on the chromosome of *P. umsongensis* GO16. Significant proteins refer to proteins that are upregulated (blue) or downregulated (pink) in the TtdR knock-out strain. LTTRs are marked in green. Acyl T, acyltransferase; MmsA, CoA-acylating methylmalonate-semialdehyde dehydrogenase; MmsB, 3-hydroxyisobutyrate dehydrogenase; KynB, arylformamidase; KynA, tryptophan 2,3-dioxygenase; TtuE, pyruvate kinase; TtuD, hydroxypyruvate reductase; GlxR, tartronate semialdehyde reductase; Hyi, hydroxypyruvate isomerase; Gcl, glyoxylate carboligase; ACS, acetyl-CoA synthetase; chemotaxis, methyl-accepting chemotaxis protein; AldB-I, aldehyde dehydrogenase; PhnX, phosphonoacetaldehyde hydrolase; MFS, MFS transporter; AEPT, 2-aminoethylphosphonate--pyruvate transaminase; OR, FAD-dependent oxidoreductase; RidA, RidA family protein; HP, hypothetical protein; Hydrolase, carbon-nitrogen hydrolase family protein; PUP, purine permease; transporter, polyamine ABC transporter substrate-binding protein; RpoZ, DNA-directed RNA polymerase subunit omega; SpoT, guanosine-3',5'-bis pyrophosphate 3'-pyrophosphohydrolase; SDR, SDR family oxidoreductase; ExbB, tonB-system energizer; ExbD, TonB system transport protein; TonB, energy transducer; recG, ATP-dependent DNA helicase RecG.

**Table S1** List of primers used for ttdR gene knockout.

| Primer | Sequence (5'-3') | Tm [°C] |
| --- | --- | --- |
| Pknock_F | CTGCAGGAATTCGATATCAAGCTTATC | 65 |
| Pknock_R | GGATCCACTAGTTCTAGAGCGG | 66 |
| ttdR_US_F | GCTCTAGAACTAGTGGATCCGTTAGCTCGGCAAACTC | 68 |
| ttdR_US_R | AAAGGGGATGCATCGATACCTCATGTATCGGCAG | 68 |
| ttdR_DS_F | GGTATCGATGCATCCCCTTTAAAATATGATTCGGGGTTG | 66 |
| ttdR_DS_R | TTGATATCGAATTCCTGCAGGATGGGATTAGCCGAAGTAGAGTATTC | 68 |
| Pknock_cPCR_F | GGAACACTTAACGGCTGACATGGG | 65 |
| Pknock_cPCR_R | CGAGGTCGACGGTATCGATAAGCTTGATATC | 66 |
| ttdR_Flank_US_F | CTTATGATCGTCCGGAATTGTTCC | 62 |
| ttdR_Flank_DS_R | GGAACGTCTTGAAAGGCACTCTC | 59 |
| PgRNA_R | CTGAGCTAGCTGTCAAAGATCTTTAGAATTC | 59 |
| PgRNA_F | CTCGAGTCTAGACTGTAGGCTTCCT | 60 |
| PgRNA_439bp_F | GTTATCAACTTGAAAAAGTGGCACCG | 59 |
| PgRNA_439bp_R | GCCTACAGTCTAGACTCGAGTAAGGAT | 60 |
| ttdR_sgRNA_20bp_F | GGGGTGACTCCGGCGATCCT | 65 |
| ttdR_Oligo118bp | CTTTGACAGCTAGCTCAGTCCTAGGTATAATACTAGTGGGGTGACTCCGGCGATCCTGTTTTAGAGCTAGAAATAGCAAGTTAAAATAAGGCTAGTCCGTTATCAACTTGAAAAAGTG |  |
| ttdR_pgRNA_cPCR_F | GGTATAATACTAGTGGGGTGACTCC | 57 |
| In_ttdR_F | CAGTTTTATTCAGGTCGCCCATAAG | 60 |
| In_ttdR_R | CCTCTCCCAGTAATCCGGAGAGTTA | 59 |

**Table S2** Primers used for pBT’T plasmid construction.

| **Primers** | **Sequence (5'-3')** | **Tm [°C]** |
| --- | --- | --- |
| pBTT_linear_F | GAATTCCTCGAGTCTAGAGGAGC | 57 |
| pBTT_linear_R | GGTACCTCCTGTTTCCTGTGTG | 60 |
| pBTT_eYFP_F | GAAACAGGAGGTACCATGGTGAGCAAG | 62 |
| pBTT_eYFP_R | GACTCGAGGAATTCTTACTTGTACAGCTC | 59 |
| pBTT_ttdR_F | CAGGAAACAGGAGGTACCATGGGTCGTTATGTCGAG | 67 |
| pBTT_ttdR_R | CTCTAGACTCGAGGAATTCTTAAAGGGGATGGGGCCTC | 67 |

**Table S3** Significant proteins from proteomics analysis. Samples were grown on 30 mM EG and 20 mM TA as substrates under nitrogen-full condition (1 g L^-1^ NH_4_Cl) and collected at 24 hours and 48 hours. A logFC (log_2_ fold change) threshold of +1 or higher indicates the protein is at least 2-fold more abundant in GO16 than in ΔttdR, whereas a logFC of -1 or lower indicates the protein is at least 2-fold more abundant in ΔttdR than in GO16. Significant proteins were selected by fold change greater than the threshold 1.5. Proteins more abundant in GO16 are colored red, and those more abundant in ΔttdR are marked in blue.

| **Locus_tag** | **Protein name** | **logFC** | **P.Value** | **adj.P.Val** | **Time** |
| --- | --- | --- | --- | --- | --- |
| F6476_RS01220 | **Gcl** / Carboxylate ligase | 10.472 | 0.00000 | 0.0029 | 48hr |
| F6476_RS01200 | **TtuE** / Pyruvate kinase | 7.085 | 0.00000 | 0.0029 | 48hr |
| F6476_RS32195 | lipocalin family protein | 5.273 | 3.34E-07 | 0.0003 | 24hr |
| F6476_RS30235 | **RecG** / ATP-dependent DNA helicase | 4.690 | 0.00001 | 0.0080 | 24hr |
| F6476_RS01215 | **Hyi** / Hydroxypyruvate isomerase | 3.838 | 0.00002 | 0.0099 | 48hr |
| F6476_RS01210 | **GlxR** / Tartronate reductase | 3.633 | 0.00002 | 0.0099 | 48hr |
| F6476_RS19665 | **PedH** / PQQ-dependent alcohol dehydrogenase | 3.184 | 0.00026 | 0.0336 | 24hr |
| F6476_RS22230 | **PhaC** / class I poly(R)-hydroxyalkanoic acid synthase | 3.037 | 0.00006 | 0.0185 | 24hr |
| F6476_RS25815 | DUF2817 domain-containing *meta*llopeptidase | 2.671 | 0.00000 | 0.0029 | 48hr |
| F6476_RS19730 | PQQ-dependent catabolism-associated CXXCW motif protein | 2.404 | 0.00046 | 0.0437 | 24hr |
| F6476_RS28575 | 3-keto-5-aminohexanoate cleavage protein | 2.364 | 0.00017 | 0.0296 | 24hr |
| F6476_RS05075 | **HrpA** / ATP-dependent RNA helicase | 2.191 | 0.00087 | 0.0441 | 24hr |
| F6476_RS00560 | **KynA** / tryptophan 2,3-dioxygenase | 1.666 | 0.00078 | 0.0441 | 24hr |
| F6476_RS32375 | **PhaC** / class II poly(R)-hydroxyalkanoic acid synthase | 1.587 | 0.00016 | 0.0296 | 24hr |
| F6476_RS26195 | NAD(P)/FAD-dependent oxidoreductase | -1.747 | 0.00040 | 0.0430 | 24hr |
| F6476_RS01855 | NO-inducible flavohemoprotein | -1.851 | 0.00102 | 0.0486 | 24hr |
| F6476_RS28945 | Hpt domain-containing response regulator | -2.059 | 0.00005 | 0.0175 | 48hr |
| F6476_RS07650 | **PhnX** / phosphonoacetaldehyde hydrolase | -2.747 | 0.00045 | 0.0437 | 24hr |
| F6476_RS22200 | TIGR01841 family phasin | -5.245 | 2.36E-07 | 0.0003 | 24hr |

**Table S4** Significant proteins from proteomics analysis. Samples were grown on 30 mM EG and 20 mM TA as substrates under nitrogen-limited condition (0.25 g L^-1^ NH_4_Cl) and collected at 24 hours and 48 hours. A logFC (log_2_ fold change) threshold of +1 or higher indicates the protein is at least 2-fold more abundant in GO16 than in ΔttdR, whereas a logFC of -1 or lower indicates the protein is at least 2-fold more abundant in ΔttdR than in GO16. Significant proteins were selected by fold change greater than the threshold 1.5. Proteins more abundant in GO16 are colored red, and those more abundant in ΔttdR are marked in blue.

| **Locus_tag** | **Protein name** | **logFC** | **P.Value** | **adj.P.Val** | **Time** |
| --- | --- | --- | --- | --- | --- |
| F6476_RS19690 | **PedE** / PQQ-dependent dehydrogenase | 5.121 | 0.00042 | 0.0474 | 24hr |
| F6476_RS10050 | 4-hydroxybenzaldehyde dehydrogenase | 4.162 | 0.00001 | 0.0077 | 48hr |
| F6476_RS07645 | **AldB-I** / aldehyde dehydrogenase | 4.048 | 0.00027 | 0.0392 | 24hr |
| F6476_RS04780 | glycine cleavage system protein R | 3.974 | 0.00001 | 0.0097 | 24hr |
| F6476_RS00245 | **TraA** / putative plasmid transfer protein | 3.109 | 0.00001 | 0.0097 | 24hr |
| F6476_RS18225 | Glycosyltransferase involved in cell wall bisynthesis | 2.964 | 0.00000 | 0.0051 | 48hr |
| F6476_RS19660 | **PedI** / aldehyde dehydrogenase | 2.759 | 0.00003 | 0.0171 | 24hr |
| F6476_RS02975 | Cold shock protein | 2.263 | 0.00001 | 0.0080 | 48hr |
| F6476_RS11700 | Replication region DNA-binding N-term | 2.170 | 0.00007 | 0.0202 | 48hr |
| F6476_RS03980 | Phosphate transporter | 1.933 | 0.00027 | 0.0368 | 48hr |
| F6476_RS10765 | DUF4242 domain-containing protein | 1.903 | 0.00032 | 0.0392 | 48hr |
| F6476_RS12900 | phosphoribosyltransferase family protein | 1.718 | 0.00050 | 0.0483 | 24hr |
| F6476_RS11380 | **Bdh** / 3-hydroxybutyrate dehydrogenase | -1.589 | 0.00008 | 0.0202 | 48hr |
| F6476_RS12525 | TetR family transcriptional regulator | -1.615 | 0.00005 | 0.0233 | 24hr |
| F6476_RS11905 | hypothetical protein | -1.642 | 0.00065 | 0.0483 | 24hr |
| F6476_RS00545 | **MmsB_1_** / 3-hydroxyisobutyrate dehydrogenase | -1.659 | 0.00003 | 0.0116 | 48hr |
| F6476_RS18080 | Glycosyltransferase family 25 (LPS biosynthesis protein) | -1.699 | 0.00031 | 0.0397 | 24hr |
| F6476_RS09730 | Histidine kinase / Sensor protein | -1.758 | 0.00005 | 0.0159 | 48hr |
| F6476_RS26410 | Glutamate dehydrogenase | -1.798 | 0.00036 | 0.0392 | 48hr |
| F6476_RS09920 | 3-phenylpropionate/trans-cinnamate dioxygenase ferredoxin reductase subunit | -1.855 | 0.00002 | 0.0083 | 48hr |
| F6476_RS00540 | **MmsA_1_** / (Methyl)malonate- semialdehyde dehydrogenase | -2.007 | 0.00010 | 0.0220 | 48hr |
| F6476_RS25985 | Pentapeptide repeat-containing protein | -2.208 | 0.00027 | 0.0368 | 48hr |
| F6476_RS32780 | Lipopolysaccharide kinase (Kdo/WaaP) family protein | -2.260 | 0.00012 | 0.0246 | 48hr |
| F6476_RS25445 | **MraZ** / Transcriptional regulator | -2.517 | 0.00021 | 0.0342 | 48hr |
| F6476_RS08435 | **ACS** / Acetyl-CoA synthetase | -2.661 | 0.00019 | 0.0336 | 48hr |
| F6476_RS28630 | **BetI** / transcriptional regulator | -3.121 | 0.00017 | 0.0315 | 24hr |
| F6476_RS08430 | **MmsA_2_** / (Methyl)malonate-semialdehyde dehydrogenase | -3.781 | 0.00002 | 0.0083 | 48hr |

**Table S5** Significant proteins from proteomics analysis. GO16 WT and ΔttdR were cultured on MSM_full_ with 20 mM TA for 48 hours. Significant proteins were selected by fold change greater than the threshold 1.5. Proteins more abundant in GO16 are colored red, and those more abundant in ΔttdR are marked in blue.

| **Locus_tag** | **Protein name** | **logFC** | | **P.Value** | **adj.P.Val** |
| --- | --- | --- | --- | --- | --- |
| F6476_RS21045 | TonB-dependent siderophore receptor | | 5.843 | 0.0000 | 0.0138 |
| F6476_RS21985 | SctC / Type 3 secretion system secretin, T3SS secretin | | 4.357 | 0.0000 | 0.0025 |
| F6476_RS05355 | pilus assembly protein | | 4.220 | 0.0000 | 0.0138 |
| F6476_RS22900 | GAF domain-containing protein | | 3.953 | 0.0000 | 0.0025 |
| F6476_RS06810 | PdxB / 4-phosphoerythronate dehydrogenase | | 3.688 | 0.0001 | 0.0149 |
| F6476_RS19660 | PQQ-dependent dehydrogenase, methanol/ethanol family | | 3.390 | 0.0009 | 0.0495 |
| F6476_RS28465 | Sarcosine oxidase subunit beta | | 3.337 | 0.0001 | 0.0145 |
| F6476_RS02630 | CNNM domain-containing protein | | 2.995 | 0.0000 | 0.0145 |
| F6476_RS30320 | PstA / Phosphate transport system permease protein | | 2.631 | 0.0009 | 0.0495 |
| F6476_RS32170 | DUF924 domain-containing protein | | 2.423 | 0.0000 | 0.0063 |
| F6476_RS01515 | Phospholipid/cholesterol/gamma-HCH transport system ATP-binding protein | | 2.346 | 0.0003 | 0.0287 |
| F6476_RS03870 | PdxH / Pyridoxine/pyridoxamine 5'-phosphate oxidase | | 2.129 | 0.0001 | 0.0169 |
| F6476_RS02285 | formate dehydrogenase subunit gamma | | 1.940 | 0.0002 | 0.0219 |
| F6476_RS23550 | (2Fe-2S)-binding protein | | 1.939 | 0.0004 | 0.0291 |
| F6476_RS20605 | PilZ domain-containing protein | | 1.914 | 0.0001 | 0.0169 |
| F6476_RS13320 | hypothetical protein | | 1.900 | 0.0003 | 0.0287 |
| F6476_RS03470 | methyl-accepting chemotaxis protein | | 1.867 | 0.0001 | 0.0169 |
| F6476_RS19690 | pentapeptide repeat-containing protein | | 1.847 | 0.0002 | 0.0219 |
| F6476_RS07145 | TonB-dependent siderophore receptor | | 1.832 | 0.0001 | 0.0194 |
| F6476_RS19675 | ABC-type amino acid transport substrate-binding protein | | 1.740 | 0.0001 | 0.0164 |
| F6476_RS29100 | insulinase family protein | | 1.694 | 0.0006 | 0.0412 |
| F6476_RS14320 | Polyphosphate kinase 2, PA0141 family | | 1.621 | 0.0005 | 0.0382 |
| F6476_RS06720 | alpha/beta hydrolase | | 1.610 | 0.0001 | 0.0169 |
| F6476_RS21290 | Membrane fusion protein, macrolide-specific efflux system | | -1.599 | 0.0006 | 0.0412 |
| F6476_RS21595 | Hypothetical protein | | -1.679 | 0.0005 | 0.0343 |
| F6476_RS22410 | Acetyltransferase | | -1.873 | 0.0003 | 0.0289 |
| F6476_RS11705 | AhpB / Alkyl hydroperoxide reductase | | -2.198 | 0.0002 | 0.0228 |
| F6476_RS21330 | TonB-dependent receptor | | -2.263 | 0.0007 | 0.0419 |
| F6476_RS25515 | Acyl-CoA dehydrogenase | | -2.454 | 0.0007 | 0.0419 |
| F6476_RS03315 | rRNA pseudouridine synthase | | -2.540 | 0.0003 | 0.0286 |
| F6476_RS26290 | NADH dehydrogenase | | -2.619 | 0.0003 | 0.0287 |
| F6476_RS32320 | phosphoribosyl-AMP cyclohydrolase | | -2.967 | 0.0001 | 0.0149 |
| F6476_RS08415 | Acyl-CoA dehydrogenase | | -3.106 | 0.0003 | 0.0287 |
| F6476_RS30260 | Rubredoxin-NAD+ reductase | | -3.168 | 0.0001 | 0.0149 |
| F6476_RS08425 | **MmsB_2_ /** 3-hydroxyisobutyrate dehydrogenase | | -3.493 | 0.0000 | 0.0138 |
| F6476_RS08430 | **MmsA_2_** / Malonate-semialdehyde dehydrogenase (Acetylating) | | -3.648 | 0.0000 | 0.0145 |

**Table S6** Significant proteins from proteomics analysis. GO16 WT and ΔttdR were cultured on MSM_lim_ with 20 mM TA for 48 hours. Significant proteins were selected by fold change greater than the threshold 1.5. Proteins more abundant in GO16 are colored red, and those more abundant in ΔttdR are marked in blue.

| **Locus_tag** | **Protein name** | **logFC** | **P.Value** | **adj.P.Val** |
| --- | --- | --- | --- | --- |
| F6476_RS21045 | TonB-dependent siderophore receptor | 4.958 | 0.00025 | 0.0296 |
| F6476_RS13110 | ATP-binding protein | 4.302 | 0.00012 | 0.0189 |
| F6476_RS23155 | Transcriptional regulator, BolA protein family | 4.084 | 0.00030 | 0.0318 |
| F6476_RS28465 | Sarcosine oxidase subunit beta | 3.978 | 0.00004 | 0.0133 |
| F6476_RS02980 | Hypothetical protein | 3.604 | 0.00014 | 0.0191 |
| F6476_RS18630 | Hypothetical protein | 2.764 | 0.00011 | 0.0189 |
| F6476_RS31280 | BcsC / Cellulose biosynthesis protein | 2.418 | 0.00051 | 0.0438 |
| F6476_RS07325 | Copper resistance protein B | 2.061 | 0.00035 | 0.0346 |
| F6476_RS32775 | serine/threonine protein kinase | 2.005 | 0.00022 | 0.0283 |
| F6476_RS05555 | FliH / flagellar assembly protein | 1.887 | 0.00012 | 0.0189 |
| F6476_RS14920 | FadR family or GntR family transcriptional regulator | 1.870 | 0.00003 | 0.0121 |
| F6476_RS12890 | Chaperonin GroEL | 1.862 | 0.00010 | 0.0189 |
| F6476_RS25480 | Stringent starvation protein A | 1.803 | 0.00007 | 0.0178 |
| F6476_RS06630 | Adenine phosphoribosyltransferase | 1.686 | 0.00045 | 0.0408 |
| F6476_RS19255 | TusC / Sulfurtransferase complex subunit | 1.641 | 0.00044 | 0.0408 |
| F6476_RS24380 | Starvation-inducible DNA-binding protein | 1.559 | 0.00048 | 0.0421 |
| F6476_RS05175 | Transcriptional regulator | 1.509 | 0.00023 | 0.0283 |
| F6476_RS00545 | **MmsB_1_** / 3-hydroxyisobutyrate dehydrogenase | -1.742 | 0.00009 | 0.0189 |
| F6476_RS26290 | NADH dehydrogenase | -1.951 | 0.00006 | 0.0168 |
| F6476_RS00540 | **MmsA_1_** / Methylmalonate-semialdehyde dehydrogenase | -2.260 | 0.00001 | 0.0038 |
| F6476_RS04205 | Hypothetical protein | -2.764 | 0.00001 | 0.0038 |
| F6476_RS08425 | **MmsB_2_** / 3-hydroxyisobutyrate dehydrogenase | -2.888 | 0.00032 | 0.0328 |
| F6476_RS21330 | TonB-dependent receptor | -3.522 | 0.00001 | 0.0038 |
| F6476_RS08435 | AMP-binding protein | -3.823 | 0.00000 | 0.0036 |
| F6476_RS11605 | Hypothetical protein | -3.854 | 0.00029 | 0.0318 |
| F6476_RS08415 | Acyl-CoA dehydrogenase | -4.268 | 0.00005 | 0.0151 |
| F6476_RS08430 | **MmsA_2_** / Malonate-semialdehyde dehydrogenase (Acetylating) | -5.538 | 0.00000 | 0.0004 |

**Table S7** Significant proteins from proteomics analysis. GO16 WT and ΔttdR were cultured on MSM_full_ with 26.5 mM Glucose for 48 hours. Significant proteins were selected by fold change greater than the threshold 1.5. Proteins more abundant in GO16 are colored red, and those more abundant in ΔttdR are marked in blue.

| **Locus_tag** | **Protein name** | **logFC** | **P.Value** | **adj.P.Val** |
| --- | --- | --- | --- | --- |
| F6476_RS31970 | carboxypeptidase regulatory-like domain-containing protein | 4.767 | 4E-06 | 0.0031 |
| F6476_RS29100 | insulinase family protein | 2.586 | 1E-05 | 0.0059 |
| F6476_RS06080 | hypothetical protein | 2.477 | 4E-05 | 0.0088 |
| F6476_RS09660 | DUF411 domain-containing protein | 2.406 | 2E-04 | 0.0223 |
| F6476_RS00935 | Cra / catabolite regulator, cra | 2.273 | 3E-04 | 0.0251 |
| F6476_RS11185 | DUF4142 domain-containing protein | 2.184 | 3E-05 | 0.0088 |
| F6476_RS05475 | FlgK, flagellar hook-associated protein | 2.157 | 8E-04 | 0.0414 |
| F6476_RS26190 | NAD(P)/FAD-dependent oxidoreductase | 1.880 | 7E-05 | 0.0142 |
| F6476_RS20875 | IbpA / Molecular chaperone | 1.754 | 1E-04 | 0.0170 |
| F6476_RS02430 | RlmN / Dual-specificity RNA methyltransferase | 1.736 | 5E-04 | 0.0387 |
| F6476_RS00920 | HAMP domain-containing protein | 1.621 | 1E-03 | 0.0436 |
| F6476_RS28075 | DNA-binding response regulator, NarL/FixJ family | 1.605 | 9E-05 | 0.0164 |
| F6476_RS15335 | NAD(P)-dependent oxidoreductase | 1.552 | 2E-04 | 0.0213 |
| F6476_RS03600 | Methyl-accepting chemotaxis protein | 1.546 | 7E-04 | 0.0414 |
| F6476_RS05945 | LpdA / Dihydrolipoyl dehydrogenase | -1.508 | 7E-04 | 0.0414 |
| F6476_RS32730 | AceF / dihydrolipoyllysine-residue acetyltransferase | -1.753 | 1E-03 | 0.0454 |
| F6476_RS07635 | Acs / acetate--CoA ligase | -1.951 | 8E-04 | 0.0414 |
| F6476_RS30395 | UvrD / DNA helicase II | -2.088 | 5E-04 | 0.0387 |
| F6476_RS26870 | ArsJ-associated glyceraldehyde-3-phosphate dehydronase | -2.173 | 2E-04 | 0.0214 |
| F6476_RS27235 | lytic murein transglycosylase | -2.316 | 1E-03 | 0.0454 |
| F6476_RS27590 | UDP-glucose 4-epimerase | -2.329 | 5E-05 | 0.0123 |
| F6476_RS23520 | RluC / Pseudouridine synthase | -2.531 | 2E-04 | 0.0234 |
| F6476_RS19640 | PqqB / pyrroloquinoline quinone biosynthesis protein | -2.538 | 4E-04 | 0.0374 |
| F6476_RS32895 | DUF1249 domain-containing protein | -2.607 | 2E-04 | 0.0234 |
| F6476_RS08430 | Malonate-semialdehyde dehydrogenase | -2.649 | 8E-04 | 0.0414 |
| F6476_RS28430 | Glutathione S-transferase | -2.751 | 1E-05 | 0.0053 |
| F6476_RS11380 | **Bdh** / 3-hydroxybutyrate dehydrogenase | -2.994 | 2E-04 | 0.0209 |
| F6476_RS32900 | ADP-ribose pyrophosphatase | -3.065 | 2E-04 | 0.0209 |
| F6476_RS09095 | Relaxase | -3.095 | 8E-04 | 0.0414 |
| F6476_RS26190 | NADH dehydrogenase, FAD-containing subunit | -3.138 | 6E-04 | 0.0412 |
| F6476_RS25980 | Pentapeptide repeat-containing protein | -3.227 | 5E-04 | 0.0387 |
| F6476_RS07645 | **AldB-I** / aldehyde dehydrogenase | -3.380 | 9E-04 | 0.0436 |
| F6476_RS19665 | Zn-dependent hydrolase, glyoxylase | -3.520 | 2E-04 | 0.0209 |
| F6476_RS19675 | ABC-type amino acid transport substrate-binding protein | -3.707 | 2E-05 | 0.0071 |
| F6476_RS19655 | **PedI** / aldehyde dehydrogenase | -3.790 | 6E-04 | 0.0389 |
| F6476_RS02575 | Protease inhibitor 142 family protein | -3.811 | 7E-05 | 0.0142 |
| F6476_RS03390 | CapB / Cold shock protein | -3.872 | 7E-06 | 0.0045 |
| F6476_RS19680 | **PedF** / Cytochrome c-550 | -5.960 | 8E-07 | 0.0010 |
| F6476_RS19660 | **PedH** / Alcohol dehydrogenase | -7.289 | 2E-05 | 0.0071 |
| F6476_RS19685 | **PedE** / PQQ-dependent dehydrogenase | -9.009 | 4E-07 | 0.0010 |

**Table S8** Significant proteins from proteomics analysis. GO16 WT and ΔttdR were cultured on nitrogen-limited MSM medium with 26.5 mM Glucose for 48 hours. Significant proteins were selected by fold change greater than the threshold 1.5. Proteins more abundant in GO16 are colored red, and those more abundant in ΔttdR are marked in blue.

| **Locus_tag** | **Protein name** | **logFC** | **P.Value** | **adj.P.Val** |
| --- | --- | --- | --- | --- |
| F6476_RS07430 | Hsp20/alpha crystallin family protein | 4.192 | 0.00076 | 0.0306 |
| F6476_RS09385 | hypothetical protein | 3.582 | 0.00022 | 0.0183 |
| F6476_RS11020 | Succinate semialdehyde dehydrogenase | 3.171 | 0.00006 | 0.0107 |
| F6476_RS03780 | CsbD domain-containing protein | 2.990 | 0.00018 | 0.0155 |
| F6476_RS21325 | cyclic peptide export ABC transporter | 2.913 | 0.00015 | 0.0149 |
| F6476_RS23885 | C4-dicarboxylate transport protein | 2.816 | 0.00022 | 0.0183 |
| F6476_RS12890 | Chaperonin GroEL | 2.792 | 0.00106 | 0.0348 |
| F6476_RS24575 | MFS transporter | 2.612 | 0.00140 | 0.0383 |
| F6476_RS12090 | ABC transporter ATP-binding protein | 2.537 | 0.00004 | 0.0106 |
| F6476_RS26540 | TrmA / tRNA (uridine(54)-C5)-methyltransferase | 2.375 | 0.00061 | 0.0298 |
| F6476_RS25720 | Acyloxyacyl hydrolase | 2.283 | 0.00032 | 0.0217 |
| F6476_RS07375 | GNAT family N-acetyltransferase | 2.207 | 0.00040 | 0.0259 |
| F6476_RS02780 | Putative spermidine/putrescine transport system ATP-binding protein | 2.037 | 0.00157 | 0.0409 |
| F6476_RS10020 | Hypothetical protein | 1.878 | 0.00243 | 0.0480 |
| F6476_RS3222 | Glycine betaine/proline transport system ATP-binding protein | 1.781 | 0.00026 | 0.0198 |
| F6476_RS07415 | Metallo-beta-lactamase family protein | 1.773 | 0.00118 | 0.0374 |
| F6476_RS32270 | Amino acid ABC transporter substrate-binding protein, PAAT family | 1.719 | 0.00085 | 0.0316 |
| F6476_RS28095 | nucleotidyltransferase family protein | 1.712 | 0.00017 | 0.0155 |
| F6476_RS02355 | glycine zipper 2TM domain-containing protein | 1.513 | 0.00010 | 0.0141 |
| F6476_RS18225 | Glycosyl transferase | -1.654 | 0.00067 | 0.0303 |
| F6476_RS19655 | **PedI** / aldehyde dehydrogenase | -1.657 | 0.00173 | 0.0428 |
| F6476_RS00540 | **MmsA_1_** / Methylmalonic acid semialdehyde dehydrogenase | -1.684 | 0.00003 | 0.0106 |
| F6476_RS33890 | Histidine kinase | -1.701 | 0.00001 | 0.0064 |
| F6476_RS10700 | CoA transferase subunit A | -1.718 | 0.00004 | 0.0106 |
| F6476_RS07645 | **AldB-I** / Aldehyde dehydrogenase | -1.729 | 0.00049 | 0.0284 |
| F6476_RS26805 | Uncharacterized protein | -1.826 | 0.00003 | 0.0106 |
| F6476_RS25370 | FtsZ / Cell division protein | -1.883 | 0.00002 | 0.0106 |
| F6476_RS19640 | PqqB / pyrroloquinoline quinone biosynthesis protein | -1.904 | 0.00194 | 0.0453 |
| F6476_RS22205 | DUF3141 domain-containing protein | -2.039 | 0.00249 | 0.0480 |
| F6476_RS11375 | Acetoacetyl-CoA synthase | -2.289 | 0.00030 | 0.0214 |
| F6476_RS09735 | CopC / Copper resistance protein | -2.299 | 0.00243 | 0.0480 |
| F6476_RS18355 | hypothetical protein | -2.392 | 0.00053 | 0.0287 |
| F6476_RS08430 | **MmsA_2_** / Malonate-semialdehyde dehydrogenase | -2.395 | 0.00073 | 0.0306 |
| F6476_RS04385 | XRE family transcriptional regulator | -2.637 | 0.00012 | 0.0141 |
| F6476_RS19500 | ATPase component of ABC-type sugar transporter | -2.694 | 0.00005 | 0.0106 |
| F6476_RS21660 | DsbD / Thiol:disulfide interchange protein | -2.824 | 0.00235 | 0.0480 |
| F6476_RS29705 | Amino acid ABC transporter ATP-binding protein, PAAT family | -2.918 | 0.00071 | 0.0303 |
| F6476_RS18310 | FMN-binding glutamate synthase family protein | -3.010 | 0.00103 | 0.0343 |
| F6476_RS11380 | **Bdh** / 3-hydroxybutyrate dehydrogenase | -3.261 | 0.00011 | 0.0141 |
| F6476_RS27150 | hybrid sensor histidine kinase/response regulator | -3.272 | 0.00068 | 0.0303 |
| F6476_RS32900 | ADP-ribose pyrophosphatase | -3.288 | 0.00000 | 0.0064 |
| F6476_RS04205 | Bacteriophage CI repressor helix-turn-helix domain-containing protein | -4.040 | 0.00004 | 0.0106 |
| F6476_RS33495 | CbpM / Chaperone modulatory protein | -4.082 | 0.00057 | 0.0289 |
| F6476_RS15050 | YejB / Microcin C ABC transporter permease | -4.245 | 0.00001 | 0.0064 |
| F6476_RS19685 | **PedE** / PQQ-dependent dehydrogenase | -7.414 | 0.00009 | 0.0141 |

**Figure S6 Genomic distribution of SNPs in evolved *P. umsongensis* GO16. Orange lines indicate chromosomal positions of mutations associated with methanol/alcohol oxidation, PHA biosynthesis, and global carbon regulation, illustrating a multi-level metabolic adaptation.**
